# An integrated genome-wide approach to discover deregulated microRNAs in non-small cell lung cancer: Clinical significance of *miR-23b-3p* deregulation

**DOI:** 10.1038/srep13236

**Published:** 2015-08-28

**Authors:** Shahnaz Begum, Masamichi Hayashi, Takenori Ogawa, Fayez J. Jabboure, Mariana Brait, Evgeny Izumchenko, Sarit Tabak, Steven A. Ahrendt, William H. Westra, Wayne Koch, David Sidransky, Mohammad O. Hoque

**Affiliations:** 1 Department of Pathology, Johns Hopkins University, Baltimore, Maryland, 21231 USA; 2 Department of Otolaryngology-Head and Neck Surgery, Johns Hopkins University, Baltimore, Maryland, 21231 USA; 3 Department of Urology, Johns Hopkins University, Baltimore, Maryland, 21231 USA; 4 Department of Oncology, Johns Hopkins University, Baltimore, Maryland, 21231 USA; 5 Rosetta Genomics Ltd. 10 Plaut St., Rehovot, Israel, 76706; 6 Department of Surgery, Division of Surgical Oncology, University of Pittsburgh Medical Center, Pittsburgh, PA, 15213 USA.

## Abstract

In spite of significant technical advances, genesis and progression of non-small cell lung cancer (NSCLC) remain poorly understood. We undertook an integrated genetic approach to discover novel microRNAs that were deregulated in NSCLCs. A total 119 primary NSCLCs with matched normal were analyzed for genome-wide copy number changes. We also tested a subset of matched samples by microRNA expression array, and integrated them to identify microRNAs positioned in allelic imbalance area. Our findings support that most of the identified deregulated microRNAs (*miR-21*, *miR-23b*, *miR-31*, *miR-126*, *miR-150*, and *miR-205*) were positioned in allelic imbalance areas. Among microRNAs tested in independent 114 NSCLCs, overexpression of *miR-23b* was revealed to be a significantly poor prognostic factor of recurrence free survival (HR = 2.40, P = 0.005, 95%CI: 1.32–4.29) and overall survival (HR = 2.35, P = 0.005, 95%CI: 1.30–4.19) in multivariable analysis. In addition, overexpression of *miR-23b* in H1838 cell line significantly increased cell proliferation, while inhibition of *miR-23b* in H1437 and H1944 cell lines significantly decreased cell doubling time. In summary, integration of genomic analysis and microRNA expression profiling could identify novel cancer-related microRNAs, and *miR-23b* could be a potential prognostic marker for early stage NSCLCs. Further biological studies of *miR-23b* are warranted for the potential development of targeted therapy.

Surgical resection remains to be the major therapy for stage I and II cases of non-small cell lung cancer (NSCLC) and is considered as a first line of treatment for better survival^1^,^2^. A recent report^3^ indicated that 5-year survival of 289 stage I NSCLC cases after surgical treatment was 63%. Many types of combination chemotherapy and targeted therapy have been investigated^4^. However, to date only limited response and survival benefit has been shown with any regimen^5^. The basis of these disappointing results stems from a lack of understanding of the basic biological mechanisms involved in the carcinogenesis of NSCLC. Cancer-specific molecular changes have utility not only as targets for therapy, but also as biomarkers for the determination of risk of recurrence for early-stage NSCLC^6^,^7^. Such prognostic capability may be due to the biologic significance of the alteration.

Soon after the identification of hundreds of new members of the microRNA family, it was shown that more than half of the known human microRNAs positioned in allelic imbalance areas of the cancer cells^8^. Such regions include minimal regions of loss of heterozygosity (LOH), which are thought to harbor tumor-suppressor genes (TSGs), minimal regions of amplification (MRA), which might contain oncogenes, common breakpoint regions in or near possible oncogenes or TSGs, and fragile sites (FRA). FRAs are preferential sites of sister-chromatid exchange, translocation, deletion, amplification or integration of plasmid DNA. The first evidence of involvement of microRNAs in cancer came from the finding that *miR-15a* and *miR-16–1* were down-regulated or deleted in most patients with chronic lymphocytic leukemia (CLL)^9^. *Mir-15a* and *miR-16-1* are located within the intron of a non-coding RNA gene of unknown function, called deleted in lymphocytic leukemia 2 (*DLEU2*). This gene resides in a 30 kb region at chromosome 13q14 that is deleted in >65% of CLL cases^9^, in 50% of mantle-cell lymphomas^10^, in 16–40% of multiple myeloma^11^ and in 60% of prostate cancers^12^. This observation led researchers to investigate the association of microRNA’s genomic locations and genomic regions that were involved in cancer. Strikingly, as noted above, 50% of the known microRNAs are located inside or close to fragile sites and in minimal regions of LOH, MRAs and common breakpoints are associated with cancer^8^. For example, the cluster *17–92* is located at 13q31, a region commonly amplified in lymphomas^13^; *miR-143* and *miR-145* are located at 5q33, which is frequently deleted in myelodisplastic syndromes, and *miR-142* is located 50 nucleotides from the t(8;17) breakpoint region, which involves chromosome 17 and *MYC*. This translocation juxtaposes the *MYC* gene to the *miR-142* promoter, and overexpresses an abnormal *MYC* that is associated with lymphomas and pro-lymphocytic leukemia^14^. The above information suggests that integration of allelic imbalance study using single-nucleotide polymorphisms (SNP) array and microRNA profiling in the same samples facilitates to identify deregulated microRNA (oncogene or tumor suppressor genes). Nymark *et al.* used microRNA expression profiling and array comparative genomic hybridization (CGH) data for detecting lung cancer-related microRNA markers and their targets. For instance, the gain at 12p13.31 was correlated with deregulated microRNAs and inversely correlated with target genes expressions^15^. Lazar *et al.* also integrated copy number alteration, mRNA and microRNA expression to identify NSCLC-related genes. They found *miR-944* expression was significantly correlated with copy number gains^16^. However, these studies did not demonstrate any *in vitro* assay of confirming the microRNA functions on living cancer cells.

In this study, we aimed to determine whether there were distinct patterns of copy number variations that correspond to the microRNA expression in NSCLCs. The discovery of such patterns would be useful for the identification of NSCLC-associated microRNA, focal genomic deletion or amplification and carcinogenesis pathway. We further explored the clinical significance of a panel of microRNAs in a well characterized cohort of NSCLC, and determined the biological significance of miR-23b using different *in vitro* assays.

## Results

We have employed an integrated genomic approach to identify altered genomic regions and deregulated microRNAs in NSCLC. Genome-wide copy number analysis in a well characterized cohort of NSCLC samples (Supplementary Figure S1) allow us to identify *de novo* allelic imbalance area in tumors that may underlie NSCLC tumorigenesis. Moreover, integration of these allelic imbalance areas with microRNA expression array data enabled us to select microRNA of interest for further studies.

### Detection of allelic imbalance areas and associated genes from copy number analysis

**Figure 1:**
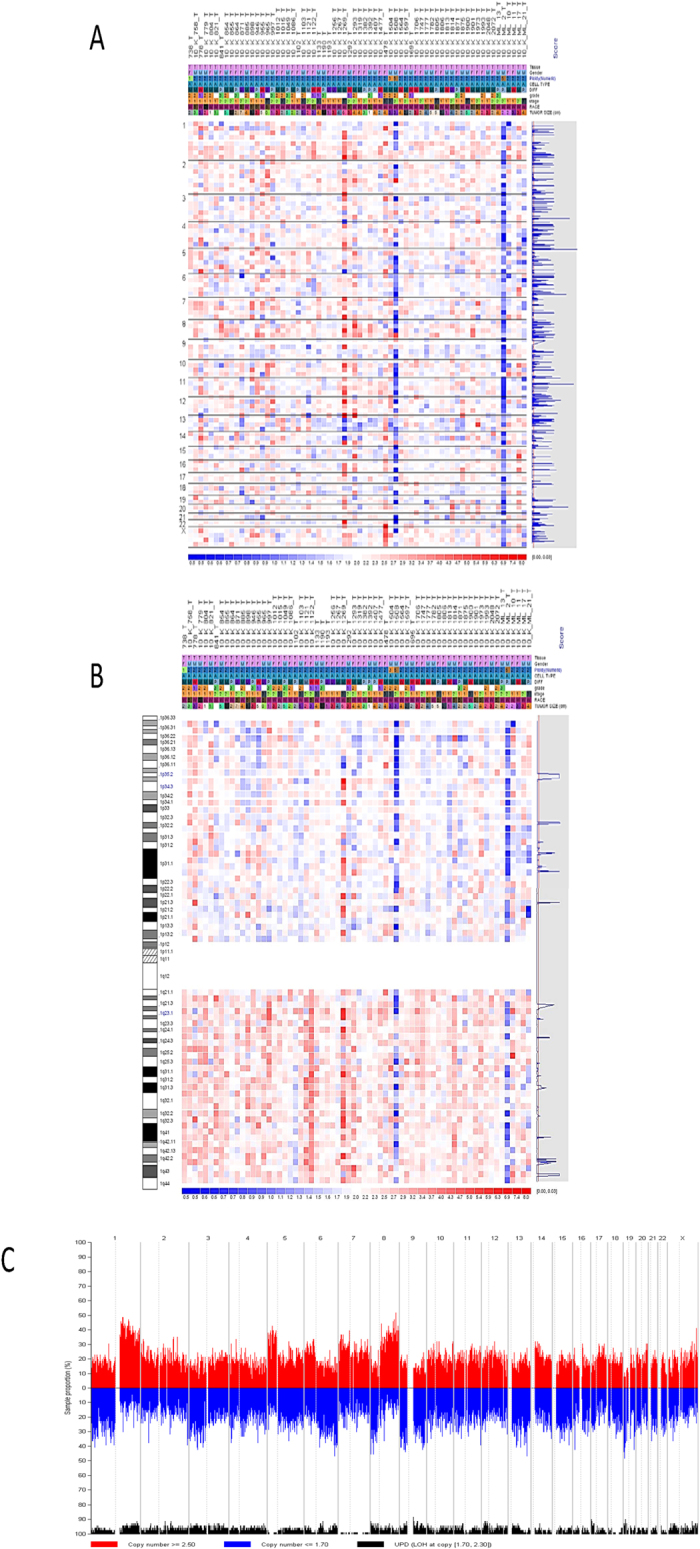
Copy number analysis of 66 ADC cases: (**A**). Copy number signals in all the chromosomal arms are displayed. The headplot at the bottom depicted the log2 ratio of copy number intensities; red color for amplifications and blue color for deletions. (**B**). More details of copy number alterations in Chromosomal arms 1p and 1q are displayed. The headplot at the bottom depicted the log2 ratio of copy number intensities; red color for amplifications and blue color for deletions. (**C**). Genome-wide copy number frequencies are plotted according to their chromosomal locations. The light grey line within each chromosome denotes the centromere, separating both p and q arms. Red color denotes copy number amplification, blue color denotes copy number deletion and black color denotes copy neutral LOH regions (somatic uniparental disomy).

An overview of copy numbers of 66 adenocarcinoma (ADC) cases in whole genome were shown in Fig. 1A. Amplified regions were shown in red color and deleted regions were indicated by blue color. In each chromosome, copy number changes were analyzed more details and correlated with approximate locus (representative example for Chr.1 is shown Fig. 1B). As evident from Fig. 1B, amplified regions are more frequent in the chromosomal arm 1q (red color) in comparison with chromosomal arm 1p (blue color). An overview of whole genome copy number pattern was shown in Fig. 1C. In our analysis, copy numbers over 2.50 were defined as amplified (upper red bar) and those under 1.70 were considered as deleted (lower blue bar). Based on the later empirical cut-off of copy numbers, amplified regions and deleted regions were shown for ADC in Fig. 1B for chromosome 1. The same procedure was also performed for 53 squamous cell carcinoma (SCC) cases (data not shown). A summary of allelic imbalanced locus that showed over 15% amplification and/or deletion in ADC and SCC are available in Supplementary Table S2. Briefly, notable amplified area identified in our analysis were: 1q21.1-1q23.3 (ADC 22.7%, SCC 16.9%) that contained potential oncogene *S100A9*; 8q21.3-22.3 (ADC 30.3%, SCC 24.5%) that contained potential oncogene *MTDH;* and 8q24.11-8q24.21 (ADC 25.8%, SCC 20.7%) that contained well known oncogene *MYC*. Some examples of key deleted loci identified by our analysis were: 4q13.1 (ADC 15.2%, SCC 22.6%) that contained potential TSG *TSGEPHA5*; 8p23.2 (ADC 18.2%, SCC 45.3%), that contained potential TSG *CSMD1* and 19q13.41-19q13.43 (ADC 18.2%, SCC 13.2%) that contained comparatively poorly characterized TSG *USP29*. In summary, in addition to confirm previously reported allelic imbalance loci such as LOH and copy number changes on chromosome 2q, 4q, 5q, 8p, 8q, 9p22 and 19q13, we identified novel LOH on 1q42 and copy number amplification on 1q21.1 and 1q32-q42 that contain some cancer associated genes that may have role in NSCLC tumorigenesis.

### Deregulated microRNAs determined by microRNA expression array

**Figure 2:**
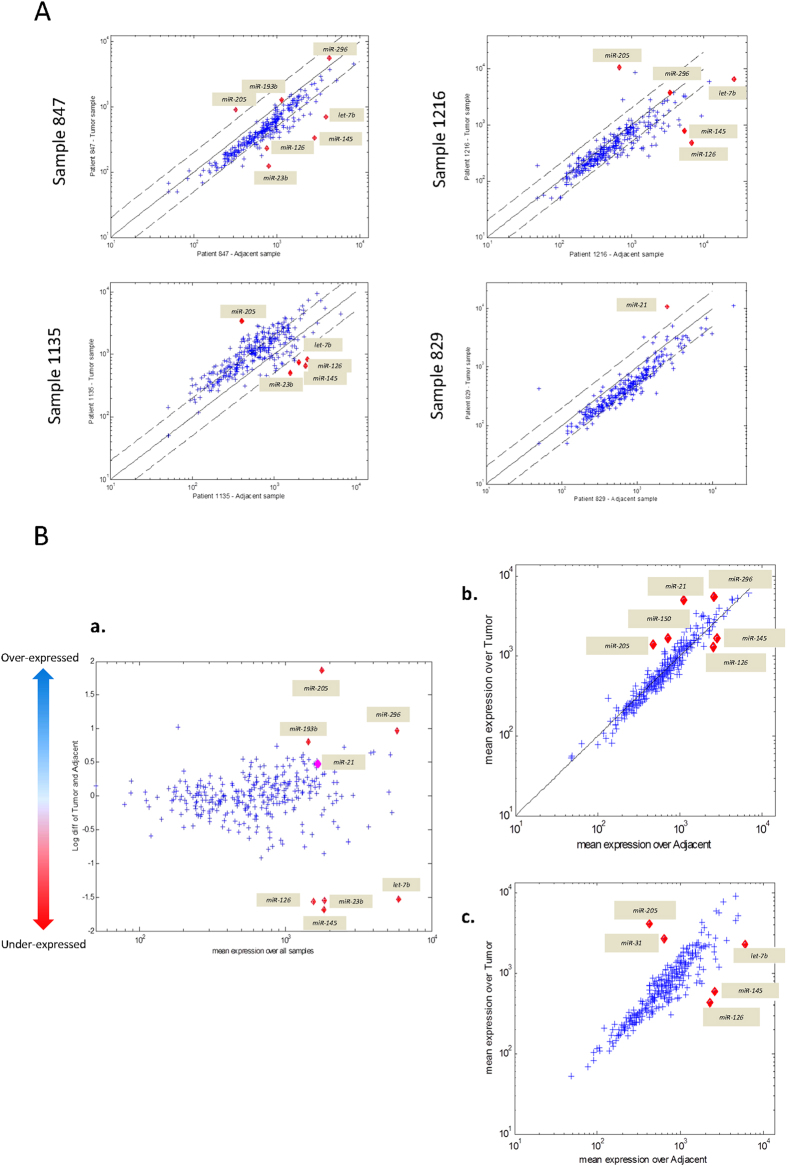
MicroRNA expression array results: (**A**). MicroRNA expression array results of representative 4 tumor-normal paired samples. Significantly differential expression between tumors and adjacent normal samples were found in some of microRNAs (red dots). (**B**). Scatter plots of promising microRNA that differentially expressed between tumors and normal by array analysis are shown: (**a**) Average microRNA expression ratios between tumor and adjacent normal tissue of all 8 cases (y axis) are plotted according to the mean expression (x axis). Differentially expressed microRNAs are expected to deviate from the bulk population (red dots). (**b**) Sub-group analyses scatter plot of mean tumor expression levels (y axis) and adjacent normal expression levels (x axis) of individual microRNAs for 3 Adenocarcinoma cases and (**c**) same for 5 squamous cell carcinoma cases. Probes with a large differential expression are identified as red dots.

Primary NSCLC and corresponding normal tissues were analyzed in 8 pairs of samples. Supplementary Table S3 summarized the clinicopathological information of all the 8 cases. The microRNA array we used here contained 688 mature microRNAs. Two types of analysis were carried out to identify probes which showed differential expression between tumor and adjacent normal tissues. Firstly, the expression ratios of individual microRNA between tumor and adjacent normal tissue were compared. Representative scatter plots are shown in Fig. 2A for 4 cases. Secondly, mean expression ratio profile across all patients was compared to the mean adjacent normal expression profile, and the differentially expressed microRNAs are shown in Fig. 2B a. In both analyses, differentially expressed microRNAs were expected to deviate from the bulk population. Four overexpressed (*miR-21*, *miR-193b*, *miR-205*, *miR-296*) and 4 under expressed microRNAs (*miR-126*, *miR-23b*, *miR-145* and *let-7b*) in tumor samples were identified by comparing mean expression values between tumors and normal (Fig. 2B a). Differential expression patterns were also observed in ADC or SCC specific manner (Fig. 2B b,c). Both ADCs and SCCs showed up-regulation of *miR-205* and down-regulation of *miR-126* and *miR-145* in tumors. Up-regulations of *miR-21*, *miR-150* and *miR-296* were only found in ADCs, whereas up-regulation of *miR-31* and down-regulation of *let-7b* were only seen in SCCs. In summary, considering mean expression values in all the 8 NSCLCs samples and group of ADC and SCC, 5 over-expressed (*miR-21*, *miR-31*, *miR-150*, *miR-205*, *miR-296*) and 3 under expressed microRNAs (*miR-126*, *miR-23b*, *miR-145*) in tumor samples were determined for technical validation.

### Technical validation of microRNA expression data by quantitative reverse transcription PCR (Q-RT-PCR) and determination of cutoff values using a training set

The comparative data between microRNA array results and Q-RT-PCR data are available in Supplementary Table S4 and S5. The individual consistency of the two techniques in representative 4 cases were high [*miR-205*:100% (3/3), miR-296:100% (2/2), miR-21:100% (1/1), miR-23b:50% (1/2), miR-126:100% (3/3), and miR-145:67% (2/3)] (Supplementary Table S4). Supplementary Table S5 summarizes the technical validation data of all the tested miRNAs in 8 tumor-normal paired samples.

**Figure 3:**
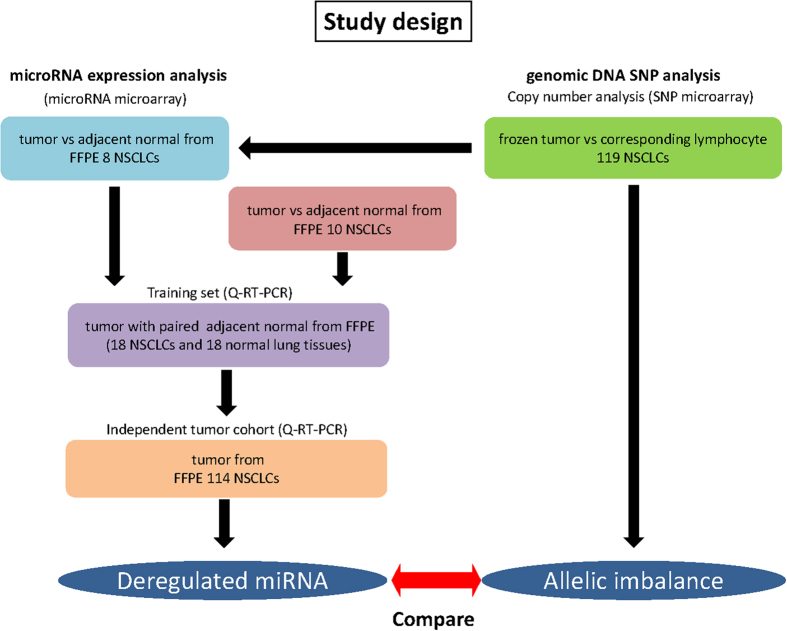
An overview of the study designs. For microRNA expression analysis, we used 3 sample cohort, technical validation set (n = 8), training set (n = 18) and independent tumor set (n = 114). For SNP analysis, we used 119 NSCLC cohorts that contain majority of samples we used for microRNA analysis. Integration of deregulated microRNAs and allelic imbalance results were performed in a subset of sample.

**Figure 4:**
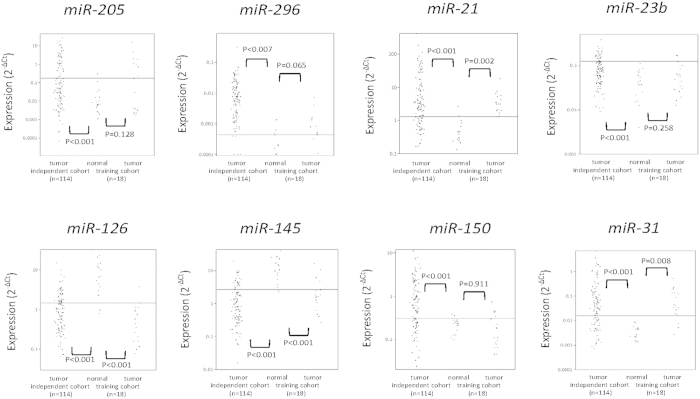
Scatter plots of 8 microRNA expression by quantitative-RT-PCR: Individual microRNA expression of independent tumor cohort (n = 114), 18 tumor-adjacent normal paired samples (training set) are shown as delta Ct values (reference gene: *miR-16*). The solid line shows empiric cut-off value which is generated by maximizing sensitivity and specificity of each of microRNA on individual ROC curves. Each tumor cohort of training set (n = 18) and independent set (n = 114) was compared with normal sample set (n = 18) by student’s t-test, two-tailed. P < 0.05 were considered as significant.

Subsequently we tested additional 10 tumor and normal paired samples by Q-RT-PCR that results in matched 18 tumor-normal cohort as a training set (n = 18). An overview of experimental design is shown in Fig. 3. Expression patterns of the training set (18 paired tumor-normal) are available in Supplementary Table S6, and scatter plots of tested miRNA by Q-RT-PCR are shown in Fig. 4. An optimal cut-off point with maximal sensitivity and specificity was determined by generating ROC curve for individual microRNA marker (solid line in Fig. 4). Using the cut-off value, the frequency of each deregulated microRNA in the tumor was as follows: 44% (8/18) overexpressed for *miR-205* (P = 0.018), 56% (10/18) overexpressed for *miR-296* (P = 0.035), 89% (16/18) overexpressed for *miR-21* (P < 0.001), 22% (4/18) overexpressed for *miR-23b* (P = 0.104), 89% (16/18) under expressed for *miR-126* (P < 0.001), 78% (14/18) under expressed for *miR-145* (P < 0.001), 33% (6/18) overexpressed for *miR-150* (P = 0.229) and 83% (15/18) overexpressed for *miR-31* (P < 0.001). A detailed summary of Q-RT-PCR analysis in training set is available in Table 1.

### Clinical validation of the microRNA panel in an independent cohort

All the 8 microRNAs analyzed in training set, were also tested in an independent cohort of 114 formalin fixed paraffin embedded (FFPE) NSCLC samples. Detailed characteristics of all these 114 NSCLCs are available in Supplementary Table S7. Among all the clinicopathological factors, only TNM stage I cases was significantly dominant in this cohort (63/114, 55.3%, P = 0.011) and the distribution of all other factors were almost similar. Delta Ct values (normalized by *miR-16*) of 114 NSCLCs were plotted in Fig. 4. Using the optimal cut off values determined in the training set, expression patterns of all the microRNAs were almost similar to those in tumors of the training set. Solid line shows empirical cut-off value of each microRNAs determined by ROC curve of training set (n = 18), and this line divides the cohort into overexpressed group and under expressed group. The frequency of each deregulated microRNA in the tumor was as follows: 39% (44/114) overexpressed for *miR-205* (P < 0.001), 95% (108/114) overexpressed for *miR-296* (P < 0.007), 71% (81/114) overexpressed for *miR-21* (P < 0.001), 23% (26/114) overexpressed for *miR-23b* (P < 0.001), 64% (73/114) under expressed for *miR-126* (P < 0.001), 78% (89/114) under expressed for *miR-145* (P < 0.001), 67% (76/114) overexpressed for *miR-150* (P < 0.001) and 67% (76/114) overexpressed for *miR-31* (P < 0.001) (Table 1).

### Association of microRNA expression with clinicopathological factors

We analyzed whether there is any association of each microRNA expression with all the available clinicopathological parameters (Supplementary Table S8). In the independent early stage dominant cohort (n = 114), positive smoking history was significantly associated with low level *miR-126* (P = 0.030, student’s t-test), N0 stage with high level *miR-23b* (P = 0.047) and TNM stage I with high level *miR-205* (P = 0.034). Significant expression difference between ADC and SCC was only seen for *miR-205* among 8 candidate microRNAs. SCC cell type was associated with *miR-205* overexpression (P < 0.001) as previously reported^17^,^18^. Although microRNA profile has been reported to be different between African-American and Caucasian^19^, in our study no significant difference was found in any candidate microRNA expression among these two ethnic groups.

All the 114 patients of independent cohort were available for follow-up. The mean follow-up period was 46.3 months (range 1.0–204.0 months). Table 2 presents the results of the univariate and multivariate survival analyses considering all the available clinicopathological factors and the expression level of all the tested microRNAs. In the univariate analysis, positive alcohol history (HR = 1.77, P = 0.042, 95%CI:1.02–3.21), poor differentiation (HR = 2.71, P = 0.001, 95%CI:1.51–4.69), TNM stage II-IV (HR = 2.45, P < 0.001, 95%CI:1.44–4.21) and high *miR-23b* expression (HR = 1.87, P = 0.028, 95%CI:1.07–3.18) were significantly associated with poor recurrence free survival (RFS). The multivariate analysis confirmed that patients with SCC histology (HR = 1.92, P = 0.017, 95%CI:1.13–3.26), TNM stage II-IV (HR = 2.74, P < 0.001, 95% CI:1.60–4.75), low *miR-150* expression (HR = 1.92, P = 0.034, 95%CI:1.05–3.47) and patients with high *miR-23b* expression (HR = 2.40, P = 0.005, 95%CI:1.32–4.29) have independently high risk of recurrence. We then explored the association of clinicopathological factors with overall survival (OS) of patients. By univariate analysis, SCC histology (HR = 2.16, P = 0.006, 95%CI: 1.26–3.72), poor differentiation (HR = 2.42, P = 0.042, 95%CI: 1.34–4.21), TNM stage II-IV (HR = 2.11, P = 0.006, 95%CI: 1.24–3.63) and high *miR-23b* expression (HR = 1.93, P = 0.019, 95%CI: 1.12–3.30) were significantly associated with poor OS. In multivariable analysis, the significant factors were SCC (HR = 2.16, P = 0.006, 95%CI: 1.26–3.72), TNM stage II-IV (HR = 2.41, P = 0.002, 95% CI: 1.40–4.22), low *miR-150* expression (HR = 1.94, P = 0.038, 95%CI:1.04–3.56) and high *miR-23b* expression (HR = 2.35, P = 0.005, 95% CI: 1.30–4.19). We also examined these factors in TNM Stage I cases (n = 63) (Table 3). Although many of factors turned out to be non-significant, histological SCC and high *miR-23b* expression were still significant prognostic factors in multivariable analysis of both recurrence free survival (RFS) and overall survival (OS). Especially, *miR-23b* was the solely significant miR factor of RFS (HR = 2.46, P = 0.041, 95% CI:1.04–5.62) and OS (HR = 2.64, P = 0.021, 95%CI:1.16–5.85) in this cohort of NSCLCs.

**Figure 5:**
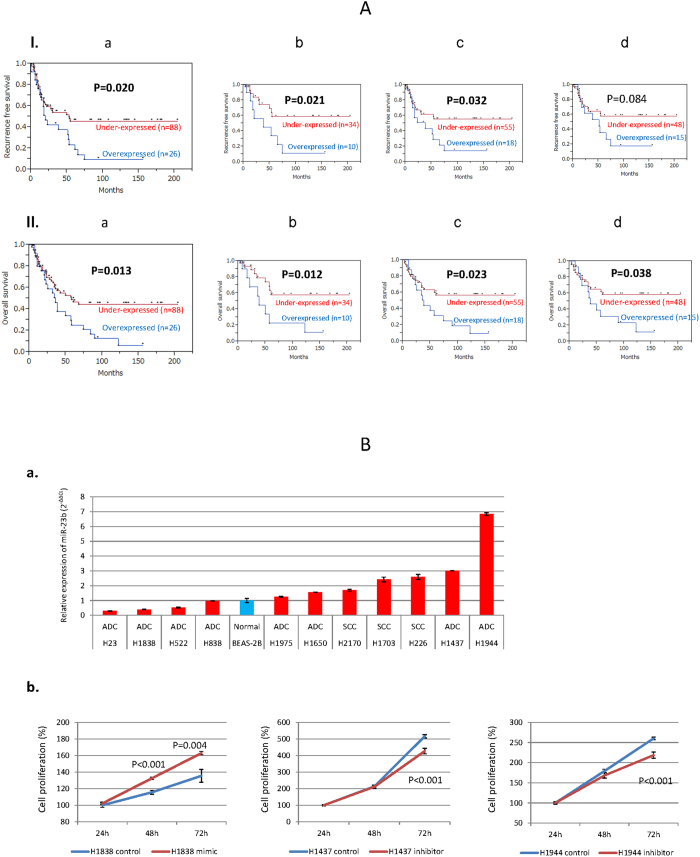
Association of clinical outcome with miR-23b expression in NSCLC and its *in vitro* oncogenic potential: (**A**). **I:** Correlation of *miR-23b* expression with recurrence free survival (RFS): Total 114 NSCLCs are divided into *miR-23b* overexpressed (n = 26) and under expressed group (n = 88): (**a**) The association of *miR-23b* expression with RFS of NSCLC patients was analyzed by Kaplan-Meier curve and log-rank test. Subgroup analyses were also performed in (**b**) T1 stage cases (n = 44), (**c**) N0 stage **c**ases (n = 73) and (**d**) TNM stage I cases (n = 63). **II:** Overall survival (OS) analysis based on *miR-23b* expression by Kaplan-Meier curve and log-rank test: (**a**) The association of *miR-23b* expression with OS of NSCLC patients was examined. Subgroup analyses were also performed in (**b**) T1 stage cases (n = 44); (**c**) N0 stage **c**ases (n = 73) and (**d**) TNM stage I cases (n = 63). P < 0.05 were considered as significant. (**B**). Modulation of *miR-23b* in lung cancer cell lines to determine its *in vitro* cell growth potential: (**a**) Relative expression (mean ± standard error) of *miR-23b* in 11 lung cancer cell lines (red bars). BEAS-2B, a normal lung epithelial cell line (blue bar) was used as a reference. ADC: Adenocarcinoma, SCC: Squamous cell carcinoma. Three adenocarcinoma cell lines were chosen to modulate *miR-23b* to understand the biological effect of this microRNA. (**b**) *MiR-23b* mimic was transfected to H1838 which showed low level *miR-23b* expression among the cell lines, while *mir-23b* inhibitor was transfected to H1437 and H1944 cell lines that showed high level *miR-23b* expression among the cell lines. Average cell proliferation ratio ± standard error was shown at each time point. P values were shown if there was significance between transfected cells and controls (student t-test, two-tailed).

As *miR-23b* overexpression was correlated significantly with poor RFS and OS, we further performed Kaplan-Meier curves analysis for *miR-23b* and associated clinical factors. Kaplan-Meier curves of RFS and OS in relation with *miR-23b* expression were shown in Fig. 5A. In log-rank test, overexpression of *mir-23b* correlated significantly with poor RFS (P = 0.020) and poor OS (P = 0.013) (Fig. 5A a). These findings remained statistically significant in subgroup analysis adjusting for T1 stage (RFS: P = 0.021, OS: P = 0.012) (Fig. 5A b), N0 stage (RFS: P = 0.032, OS: P = 0.023) (Fig. 5A c) and TNM stage I (OS: P = 0.038) (Fig. 5A d).

### MicroRNA expression and allelic imbalance

We integrated allelic imbalance area from copy number analysis cohort (n = 119) (Fig. 3) with loci of all the 8 microRNAs we tested in this study. We considered allelic imbalance loci if a SNP locus is amplified or deleted at least in 15% of our tested samples. The integrated data of copy number variation (CNV) determined by SNP array analysis and deregulated candidate microRNAs were shown in Table 4. Four out of 8 deregulated microRNAs were located in gene locus with over 15% allelic imbalance (amplification or deletion). Among 119 NSCLCs, the locus of *miR-205* showed amplifications in 29/119 (24.4%) cases and deletions in 13/119 (10.9%) cases. The locus of *miR-126* showed amplifications in 11/119 (9.2%) cases and deletions in 22/119 (18.5%) cases. The locus of *miR-150* showed amplifications in 10/119 (8.4%) and deletions in 19/119 (16.0%) cases. The locus of *miR-31* showed amplifications in 6/119 (5.0%) and deletions in 20/119 (16.8%) cases. The locus of rest of the microRNAs (*miR-296*, *miR-21*, *miR-23b* and *miR-145*) had no or low level of allelic imbalance.

### Overexpression and inhibition of miR-23b in lung cancer cell lines

To determine the biological consequences of *miR-23b* deregulation, we first analyzed the expression level of *miR-23b* in a panel of NSCLC cell lines and one immortalized normal broncho-epithelial cell line (Fig. 5B a). We decided to perform functional analysis using cell lines that were derived from ADCs because of the wide variability of expression levels. We selected H1838 cell line for *miR-23b* overexpression (mimic), and H1437 & H1944 cell lines for inhibition of *miR-23b*. We performed [3-(4,5-dimethylthiazol-2-yl)-2,5 diphenyl tetrazolium bromide] (MTT) assay using these three cells either overexpressing or inhibiting *miR-23b*. In the mimic study, cell viability was significantly increased in *miR-23b* transfected H1838 cells than control at 72 hours after transfection (p < 0.001, student t-test). While in the inhibition study, *miR-23b* siRNA transfected H1437 and H1944 cells showed significant decrease in cells viability compared to the control at 72 hours after transfection (p < 0.001, student t-test) (Fig. 5B b). These phenotypic characteristics due to forced alterations of *miR-23b* were consistent with primary NSCLC data that *miR-23b* is a potential oncogene.

## Discussion

Generally, recurrent losses and gains of broad chromosomal regions in cancer suggest that multiple genes located in the same chromosomal regions may be concurrently function as a tumor suppressor gene (TSG) and oncogene. In addition to the identification of novel allelic imbalance area, our data provided confirmation of previously reported allelic imbalance area that may contain novel NSCLC related cancer genes. Furthermore, the integrated approach we employed in this study support the previous assumption that almost half of microRNA are located in the allelic imbalance areas.

NSCLC is an extensively heterogeneous disease at the molecular level which in part may be related to easy exposure of lung to different kinds of environmental stimuli. Various environmental stimuli and individual response to a particular stimuli leads to differential molecular alterations that include mutations, translocations, copy number alterations etc. All these alterations may have an impact on NSCLC initiation and/or progression and may be targeted for therapy and the development of preventive strategies. Comprehensive investigation of focal copy number alterations in NSCLC has led to the identification of multiple cancer-driving genes with potential therapeutic implications^20^,^21^,^22^. However, all these approaches for identification of genetic alterations have limitations and therefore development of novel analytical approaches may generate new, previously not discovered targets for further studies. By our analysis, we have identified novel regions of allelic imbalance that contain several cancer related genes such as *CHD1L* and *S100A9* at locus 1q21.1; and *CENPF* and *ESRRG* at locus 1q32-q42. However, since the main focus of this study was to identify NSCLC related microRNAs, we yet not further validated and functionally characterized any of these genes for potential clinical implications.

We have identified several differentially expressed microRNAs in cancer and matching normal samples of NSCLC. Some of our findings are consistent with previously reported data while others are inconsistent. As for example, *miR-31* was reported to be overexpressed in colorectal cancer^23^ which is consistent with our finding; however, it was also reported to be under expressed due to promoter hypermethylation in prostate cancer^24^. These contradictions could be due to tumor context, technology used for analysis and different endogenous and exogenous stimuli for the genesis of a given tumor in a given population. Other microRNAs that were overexpressed in our study such as *miR-21*, *miR-205* and *miR-296* were also reported to be deregulated in other cancer types. As for example, *miR-205* was reported to be under expressed in non-muscle invasive urothelial cancer, however it was found to be overexpressed in muscle-invasive urothelial cancer^25^. Similarly in consistent with our results, *miR-126* and *miR-145* were also reported to be under expressed in NSCLC^26^,^27^, and functionally *miR-145* was revealed to inhibit NSCLC cell proliferation by targeting a well-known oncogene, c-Myc^28^. However, contradictory findings were also reported for *miR-145*^29^. In the later study, although *miR-145* was under expressed in lung cancer, overexpression of *miR-145* was related to poor survival. Thus, detail biological studies are necessary for understanding the exact role of *miR-145* in the genesis of NSCLC.

Our array analysis data indicate that *miR-23b* is under expressed in NSCLC (Fig. 2B a). However, in our independent cohort study by Q-RT-PCR (Fig. 4), we found that *miR-23b* was overexpressed in NSCLC. These differences may derive from the fact that the array result only showed the total average of 8 samples. Total average is not necessarily indicates whether the overexpressed or under expressed cases are dominant. Actually, each of subgroup analysis (3ADCs and 5 SCCs) of *miR-23b* did not show either overexpression or under expression (Supplementary Table S5) which is also supported by Q-RT-PCR results of training set (n = 18) (Supplementary Table S6). Another explanation is the existence of dual role of this microRNA as described above for *miR-205*. If the function of a given microRNA differs in each tumor stage, the expression pattern will depend on the characteristics of the cohort. Actually, *miR-23b* expression was significantly different between pathological N0 stage and N1/N2 stages (P = 0.047, student t-test, Supplementary Table S8). Both overexpression and under expression of *miR-23b* were reported previously in various solid tumors^30^. Our independent tumor cohort (n = 114) mainly consisted of TNM stage I tumors (63/114, 55.2%), and it is slightly different from array cohort (3/8, 37.5%) (Supplementary Table S7).

The integration of allelic imbalance determined by SNP array and our limited microRNA expression data support that half of the microRNAs are positioned in allelic imbalance areas. Although loci imbalance status of *miR-126* and *miR-150* (Table 4) showed good correlation with microRNA expression by Q-RT-PCR (Supplementary Table S5, S6), the expression pattern of *miR-31* and *miR-205* didn’t match their loci imbalance status. Possible explanations underlying these inconsistencies are existence of another deregulating mechanism, inadequate threshold setting or array noises. Meanwhile, in order to find low level allelic imbalance, we manually analyzed SNP data in the locus of *miR-21* and *miR-23b* using the same threshold (amplification >2.5, deletion <1.7). Expressions of both of these microRNAs were overexpressed in tumors by Q-RT-PCR analysis (Fig. 4). We then extended our analysis in all the SNP data set (n = 119) and as expected observed one of previously well characterized microRNA, *miR-21* locus showed significant amplification in tumor (Supplementary Table S9), and our focus *miR-23b* locus was also showed significant amplification in tumor (Supplementary Table S10). Overexpression of *miR-21* and *miR-23b* in NSCLCs seemed to be partially due to allelic amplification.

While it is evident that *miR-23b* is a potential oncogene for the genesis and progression of NSCLC by our initial functional analysis data and testing of NSCLC samples, *miR-23b* has been found to have dual role in carcinogenesis, both as a tumor suppressor and as an oncomir. A detailed review of functional consequences by *miR-23b* has been published recently^30^,^31^. As a tumor suppressor, Majid *et al.*^31^ reported that *miR-23b* was frequently silenced in prostate cancer by methylation, and it had anti-proliferative and anti-invasive properties through repressing Src kinase/Akt pathway. Promoter hypermethylation of *miR-23b* was also found in gliomas^32^. Another recent report suggested that radio-resistant pancreatic cancer cell lines showed the reduced levels of *miR-23b*, and overexpression of *miR-23b* sensitized the cells to radiation by targeting ATG12, a known autophagy-related protein^33^. On the contrary, Jin *et al.* indicated the oncogenic character of *miR-23b* in breast cancer, showing that ERBB2, EGF and TNF-α promote its expression through AKT/NF-κB pathway^34^. Chen *et al.* revealed that down-regulation of *miR-23b* was followed by the inhibition of β-catenin/Tcf-4 and HIF-1α/VEGF signaling pathways in glioma^35^. Recently, Zaman *et al.* found that PTEN was overexpressed after *miR-23b-3p* knock-down in renal cancer cells and also found inverse correlation of *miR-23b* with PTEN expression in human samples^36^. PTEN inactivation was reported as a poor prognostic factor^37^,^38^ or a chemotherapy resistant factor^39^,^40^ for NSCLC. As a future study, we have to elucidate the gene targets of *miR-23b* in NSCLCs including PTEN^36^, and find mechanism of involvement of *miR-23b* in lung carcinogenesis.

There are several reports about the prognostic significance of miRNAs in early stage NSCLCs^41^,^42^,^43^,^44^,^45^. Yanaihara *et al* tested a panel of miRNAs in 104 NSCLCs including 65 stage I tumors (62.5%)^46^. They found *miR-205*, *miR-21* and *miR-150* were overexpressed, while *miR-126* and *miR-145* were under expressed in NSCLCs. Other groups reported overexpression of *miR-31* in NSCLCs^47^,^48^. Patnaik *et al* examined miRNA expression profiles that might predict recurrence of localized stage I NSCLC after surgery^49^. They compared 37 recurrent and 40 non-recurrent cases. Although they did not focus on *mir-23b* in their study, it was listed as one of the significantly overexpressed microRNAs in recurrent cases (P = 0.003, Fold change: 2.51). All these reports may partially support our results. In this study, we evaluated prognostic significance of miRNAs expression in early stage NSCLCs using formalin fixed paraffin embedded (FFPE) samples. If reliable prognostic markers would be available in early stage NSCLC, patients could be handled in a more appropriate way to increase survival time. In addition, miRNA marker also has potential for monitoring of disease as miRNAs are more stable than messenger RNAs due to its small size^50^, and can be tested in bodily fluids such as in sputum^51^ and serum^52^. Several studies have already been published using miRNA microarray data from FFPE samples^53^,^54^,^55^. As they are often the only available tissue source with comprehensive clinical data and long-term follow up, it is meaningful to prove their qualities. Hall *et al* showed that microRNAs were not subjected to the same deterioration seen in other RNA types^53^. Based on these reports, this study also showed that micro array results from FFPE samples were almost consistent with Q-RT-PCR results.

In conclusion, we have identified novel allelic imbalance regions that could harbor potential NSCLC related genes. Our integrated analysis revealed that a substantial numbers of microRNA were located in allelic imbalance area. More interestingly, from the clinical context, increased *miR-23b* expression in the tumor is a novel candidate biomarker of significant for poor survival of NSCLC patients. However, further validation of *miR-23b* in a multi-centered prospective study is needed before any potential clinical implementation; and detail *miR-23b* deregulation mechanisms including downstream pathway should be evaluated not only for the potential as a prognostic marker but also for suitability of early detection marker and targeted therapy of NSCLC.

## Methods

### Clinical samples

Different set of NSCLC clinical samples were tested in this study. An overview of study design was shown in Fig. 3. A total 119 patients undergoing surgical resection of a primary NSCLC at The Johns Hopkins Hospital (Baltimore, MD, USA), the Johns Hopkins Bayview Medical Center (Baltimore, MD, USA) or the Medical College of Wisconsin, Froedtert Memorial Hospital (Milwaukee, WI, USA) were included for SNP array analysis. Among these specimens, 66 were adenocarcinoma (ADC) (including those with bronchoalveolar components), and 53 were squamous cell carcinoma (SCC). Details criteria of these samples are available in Supplementary Table S1. A subset (8 pairs) of the cohort (Supplementary Table S3) was analyzed for microRNA expression array analysis, and 8 candidate microRNAs were selected for further validation based of microRNA array data bioinformatics analysis. Ten additional sample pairs were added to these 8 samples, and tested by Q-RT-PCR as a training set (n = 18) (Fig. 3). As an independent set, 114 NSCLC tumors were used for testing 8 candidate microRNAs. The clinicopathological characteristics of this independent cohort of 114 NSCLC samples were summarized in Supplementary Table S7. All samples were obtained as anonymized materials in accordance with the guidelines which were approved by the Johns Hopkins University Institutional Review Board. Thus, this study was qualified for exemption under the U.S. Department of Health and Human Services policy for protection of human subjects [45 CFR 46.101(b)].

### DNA extraction

Hematoxylin-Eosin stained sections were histologically examined at every 20 sections for the presence or absence of tumor cells, as well as for tumor density. Only sections that showed more than 70% of tumor cells were used for DNA extraction. Microdisected tissues and lymphocytes were digested with 1% SDS and 50 μg/ml proteinase K (Boehringer, Mannheim, Germany) at 48°C overnight followed by phenol/chloroform extraction and ethanol precipitation of DNA as previously described^56^.

### SNP microarray analysis

Genomic DNA from microdissected frozen tumor tissues and corresponding lymphocyte were analyzed in parallel. Briefly 250 ng DNA was digested with *Xba*I (New England Biolabs Inc., Ipswich, MA, USA), ligated to the adaptor, and amplified by polymerase chain reaction (PCR) using a single primer. After purification of PCR products with the MinElute 96 UF PCR purification kit (Qiagen, Valencia, CA, USA), amplicons were quantified, fragmented, labeled and subsequently hybridized on Affymetrix GeneChip1 Mapping 10 K 2.0 SNP microarrays following the manufacturer’s instructions (Affymetrix Inc., Santa Clara, CA, USA). After washing and staining, the arrays were scanned for data analysis.

### RNA extraction for microRNA expression analysis

Total RNA was extracted from the 10-μm-thick FFPE tissue sections as previously described^57^ using the Ambion RecoverAll Total Nucleic Acid Isolation Kit for FFPE tissues (Applied Biosystems/Ambion, Austin, TX, USA) according to the manufacturer’s instructions. Only sections that showed more than 70% of tumor cells were used for RNA extraction. The quantity and quality of the total RNA was verified with the NanoDrop spectrophotometer (Thermo Fisher Scientific, Waltham, MA, USA). Twelve pairs of tumor-adjacent samples were hybridized. Of these, 8 pairs were considered highest quality and were used for the following experiments.

### miRdicator^TM^ array platform

Custom microarrays were produced by printing DNA oligonucleotide probes representing 688 microRNAs (Sanger database, version 9 and additional Rosetta validated and predicted microRNAs). Each probe, printed in triplicate, was carried up to 22-nt linker at the 3′ end of the microRNA’s complement sequence in addition to an amine group used to couple the probes to coated glass slides. 10/20 μM of each probe were dissolved in 2× SSC + 0.0035% SDS and spotted in triplicate on Schott Nexterion® Slide E (Applied Microarrays Inc., Tempe, AZ, USA) coated microarray slides using a Genomic Solutions® BioRobotics MicroGrid II (Genomic solutions, Beverly, MA, USA) according to the manufacturer’s directions. Sixty four negative control probes were designed using the sense sequences of different microRNAs. Two groups of positive control probes were designed to hybridize for miRdicator^TM^ array (Rosetta Genomics Inc., Philadelphia, PA, USA) synthetic spikes. Small RNA was added to the RNA before labeling to verify the labeling efficiency. Probes for abundant small RNA [e.g. small nuclear RNAs (U43, U49, U24, Z30, U6, U48, U44), 5.8 s and 5 s ribosomal RNA] were spotted on the array to verify RNA quality. The slides were blocked in a solution containing 50 mM ethanolamine, 1 M Tris (pH9.0) and 0.1% SDS for 20 min at 50 ^°^C, then thoroughly rinsed with water and spun dry.

### Cy-dye labeling of microRNA for miRdicator^TM^ array

Total RNA (3–5 μg) was labeled by ligation of a RNA-linker, p-rCrU-Cy/dye (Dharmacon, Lafayette, CO, USA)^58^ to the 3′-end with Cy3 or Cy5. The labeling reaction contained total RNA, spikes (20-0.1 fmoles), 300 ng RNA-linker-dye (Dharmacon), 15% DMSO, 1x ligase buffer and 20 units of T4 RNA ligase (New England Biolabs Inc.) and proceeded at 40 °C for 1 hr followed by 1 hr at 37 °C. The labeled RNA was mixed with 3x hybridization buffer (Ambion, Austin, TX, USA), heated to 95 °C for 3 min and then added on top of the miRdicator^TM^ array. Slides were hybridize 12–16 hr in 42 °C, followed by two washes in room temperature with 1xSSC and 0.2% SDS and a final wash with 0.1xSSC. The array was scanned using an Agilent Microarray Scanner Bundle G2565BA resolution of 10 μm at 100% power) (Agilent Technologies, Santa Clara, CA, USA). The data was analyzed using SpotReader software (Niles Scientific, Seattle, WA). Standard bioinformatics and statistical analysis were performed.

### Real Time reverse transcriptase (RT) polymerase chain reaction (Q-RT-PCR) for Quantification of microRNAs

Briefly, a total of 10 ng RNA isolated from primary tissues was reverse transcribed using TaqMan reverse transcription kit (Applied Biosystems, Foster City, CA, USA) and microRNA-specific primers provided with TaqMan microRNA assays (Applied Biosystems) in 15 μL reaction volume that contains 3 μL of RT Primer Mix, 0.15 μL of 100 mM dNTPs, 1 μL of Reverse Transcriptase enzyme 50 U/μL, 0.19 μL of RNase inhibitor 20 U/μL, 4.16 μL of Nuclease Free water and 5 μL of RNA (10 ng). RT reaction was carried out with annealing at 16 ^°^C for 30 min followed by extension at 42 ^°^C for 30 min. 1.3 μL of the RT reaction was then used with 1 μL specific primers for each microRNAs (Applied Biosystems) in triplicate wells for 45-cycles PCR on a 7900HT thermocycler (Applied Biosystems). The thermal cycling parameters were as follows: 50 ^°^C for 2 min, 95 ^°^C for 10 min, followed by a third step for denaturation at 95 ^°^C for 15 s and annealing/extension at 60 ^°^C for 1 min repeated for 40 cycles. SDS v2.4 software (Applied Biosystems) was used to determine cycle threshold (Ct) values of the fluorescence measured during PCR. All experiments were done in triplicate. Two normalization steps were considered: loading the same quantity of template RNA in each well and normalizing the data against endogenous genes (*hsa-miR-16*, *RNU6*). As expression of *hsa-miR-16* was evenly distributed across the samples, we decided to use *hsa-miR-16* for normalization in this study (data not shown).The ABI TaqMan SDS v 2.4 software was utilized to obtain raw Ct values. Relative quantification of microRNA expression was calculated with the 2(-Delta Delta Ct) method (Applied Biosystems User Bulletin N 2) (P/N 10303859).

### Lung cancer cell lines

Lung cancer cell lines H23, H226, H522, H838, H1437, H1650, H1703, H1838, H1944, H1975, H2170 and SV40-Immortalized normal human bronchial epithelium cell line BEAS-2B were obtained from and propagated according to the recommendations of American Type Culture Collection (ATCC). Mediums and antibiotics were purchased from Mediatech (Manassas, VA, USA) and supplemented with fetal bovine serum (10%) (Hyclone, Logan, UT, USA), 100 μg/ml streptomycin and 100 I.U/ml penicillin (both from Life technologies). Cells were grown at 37 °C in a humidified atmosphere composed of 95% air and 5% CO_2_ in a monolayer culture. All cancer cell lines were maintained in RPMI 1640, and BEAD-2B was grown in BEGM (Lonza, Walkersville, MD, USA) medium.

### Transfection of miR-23b

We first determined the expression level of *miR-23b* in different lung cancer cell line (H23, H226, H522, H838, H1437, H1650, H1703, H1838, H1944, H1975, H2170) and one SV40-immortalized normal alveolar cell line (BEAS-2B). Based on expression patterns, we selected 3 cell lines (one expressing low level and two expressing high level of *miR-23b*) to determine the biological effect on cell lines due to *miR-23b* modulation. H1838, H1437 and H1944 cells (5 × 10^3^–10 × 10^3^ cells/well) were plated on 96-well plates with 100 μl of growth medium without antibiotics. The cells were transfected with RNAiMAX (Life technologies, Carlsbad, CA, USA) according to the manufacturer’s protocol. Both mirVana® miRNA mimic Negative Control #1 and mirVana® miRNA inhibitor Negative Control #1 (Life technologies) were used as controls. Transfection efficiency was maximized at 50 nM for mimic (H1838: 2,328, 672%) and 3.3 nM for inhibition (H1437: 0.007%, H1944: 0.010%).

### Cell Proliferation assay (MTT assay)

Transfected lung cancer cell lines were plated on 96-well plates at a density of 5 × 10^3^ to 1 × 10^4^ per well. Cellular viability was measured by the MTT proliferation assay kit (ATCC, Manassas, VA, USA) according to the manufacturer’s instructions as described in the previous paper from our group^59^. Each assay was performed in triplicate, and each experiment was repeated at least three times. The extent of cellular survival was represented as a percentage of the first measurement day.

### Data Analysis and Statistical Consideration

Allelic calls for tumor DNA and corresponding normal genomic DNA were obtained from normalized SNP array data using the GDAS genotyping software supplied by the array manufacturer (Affymetrix). A Hidden Markov Model was applied to infer the probability of allelic imbalance for each SNP in tumor DNA compared to corresponding normal DNA using the dChip software (Cheng Li Lab). Further details of identified allelic imbalance area are described in our previous study^60^.

We determined an optimal cut off value for each tested microRNA using a training set that consist of 18 tumors with paired normal. ROC curve was generated for each microRNA and the empiric cut off value was selected by maximizing sensitivity and specificity. Based on this cut off value, we divided another set of 114 tumor samples into overexpressed and under expressed groups and the differences of clinical outcomes were compared between overexpressed and under expressed groups. For clinical outcomes, RFS was defined as the time from surgery to the time of first documentation of any disease recurrence. OS was defined as the time from surgery to the time of death of disease. Those who remained alive were censored at the last date the subject was known to be alive. Associations of microRNA expressions with RFS and OS were evaluated using Cox proportional hazards model with hazard ratios and 95% confidence intervals estimated for multivariable analysis. For other statistics, continuous variables were analyzed by Student’s t-test and categorical variables were analyzed by Fisher’s exact test. All statistical analyses were performed using JMP 9 software (SAS institute, Cary, NC, USA). The level of statistical significance was set at P < 0.05 in two-tailed.

## Additional Information

**How to cite this article**: Begum, S. *et al.* An integrated genome-wide approach to discover deregulated microRNAs in non-small cell lung cancer: Clinical significance of *miR-23b-3p* deregulation. *Sci. Rep.* **5**, 13236; doi: 10.1038/srep13236 (2015).

## Supplementary Material

Supplementary Information

## Figures and Tables

**Table 1:** Frequency of high or low expression of each microRNA in the training set (n = 18) and in independent set (n = 114).

microRNA(Cut-off value)	Expression	A:Normal intraining set(n = 18)	B:Cancer intraining set(n = 18)	P value(A vs B)	C:Cancer inindependentcohort (n = 114)	P value(A vs C)
*miR-205* (0.173)	OverexpressedUnder expressed	6% (1/18)94% (17/18)	44% (8/18)56% (10/18)	**0.018**	39% (44/114)61% (70/114)	**0.006**
*miR-296* (0.00044)	OverexpressedUnder expressed	17% (3/18)83% (15/18)	56% (10/18)44% (8/18)	**0.035**	95% (108/114)5% (6/114)	**<0.001**
*miR-21* (1.297)	OverexpressedUnder expressed	11% (2/18)89% (16/18)	89% (16/18)11% (2/18)	**<0.001**	71% (81/114)29% (33/114)	**<0.001**
*miR-23b* (0.124)	OverexpressedUnder expressed	0% (0/18)100% (18/18)	22% (4/18)78% (14/18)	0.104	23% (26/114)77% (88/114)	**0.023**
*miR-126* (1.470)	OverexpressedUnder expressed	89% (16/18)11% (2/18)	11% (2/18)89% (16/18)	**<0.001**	36% (41/114)64% (73/114)	**<0.001**
*miR-145* (2.680)	OverexpressedUnder expressed	89% (16/18)11% (2/18)	22% (4/18)78% (14/18)	**<0.001**	22% (25/114)78% (89/114)	**<0.001**
*miR-150* (0.302)	OverexpressedUnder expressed	11% (2/18)89% (16/18)	33% (6/18)67% (12/18)	0.229	67% (76/114)33% (38/114)	**<0.001**
*miR-31* (0.016)	OverexpressedUnder expressed	0% (0/18)100% (18/18)	83% (15/18)17% (3/18)	**<0.001**	67% (76/114)33% (38/114)	**<0.001**

P value was calculated by Fisher’s exact test, two-tailed. If significant displayed bold.

**Table 2:** Correlation of clinicopathological and miR factors with clinical outcome in validation cohort (n = 114).

	RFS	Univariate	Multivariable	OS	Univariate	Multivariable
Age (>60 vs ≤ 60)	>60	HR = 1.02, P = 0.93595%CI:0.59–1.83		>60	HR = 1.17, P = 0.59095%CI:0.67–2.15	
Race (AA vs non-AA)	AA	HR = 1.06, P = 0.85295%CI:0.54–1.94		AA	HR = 1.14, P = 0.69295%CI:0.58–2.09	
Gender (Female vs Male)	Male	HR = 1.20, P = 0.49895%CI:0.71–2.06		Male	HR = 1.21, P = 0.47995%CI:0.71–2.09	
Smoking history (Yes vs No)	Yes	HR = 1.29, P = 0.34495%CI:0.76–2.23		Yes	HR = 1.33, P = 0.29195%CI:0.78–2.31	
Alcohol history (Yes vs No)	Yes	**HR = 1.77,** **P = 0.04295%CI:1.02**–**3.21**		Yes	HR = 1.50, P = 0.14195%CI:0.88–2.65	
Histology (SCC vs ADC)	SCC	HR = 1.61, P = 0.07595%CI:0.95–2.72	**HR = 1.92**,**P = 0.01795%CI:1.13**–**3.26**	SCC	**HR = 1.71, P = 0.04895%CI:1.01**–**2.90**	**HR = 2.16**,**P = 0.00695%CI:1.26**–**3.72**
Differentiation (Poor vs Well/Mod)	Poor	**HR = 2.71, P = 0.00195%CI:1.51**–**4.69**		Poor	**HR = 2.42, P = 0.04295%CI:1.34**–**4.21**	
TNM stage (II/III/IV vs I)	II–IV	**HR = 2.45, P < 0.00195%CI:1.44**–**4.21**	**HR = 2.74,P < 0.00195%CI:1.60**–**4.75**	II–IV	**HR = 2.11, P = 0.00695%CI:1.24**–**3.63**	**HR = 2.41,P = 0.00295%CI:1.40**–**4.22**
*miR-21* (High vs Low)	High	HR = 1.65, P = 0.09395%CI:0.92–3.14		High	HR = 1.61, P = 0.11095%CI:0.90–3.07	
*miR-23b* (High vs Low)	High	**HR = 1.87, P = 0.02895%CI:1.07**–**3.18**	**HR = 2.40,P = 0.00595%CI:1.32**–**4.29**	High	**HR = 1.93, P = 0.01995%CI:1.12**–**3.30**	**HR = 2.35,P = 0.00595%CI:1.30**–**4.19**
*miR-31* (High vs Low)	High	HR = 1.22, P = 0.48595%CI:0.70–2.21		High	HR = 1.70, P = 0.08195%CI:0.94–3.29	
*miR-126* (High vs Low)	Low	HR = 1.42, P = 0.20395%CI:0.83–2.52		Low	HR = 1.31, P = 0.32995%CI:0.77–2.31	
*miR-145* (High vs Low)	Low	HR = 1.07, P = 0.84495%CI:0.58–2.11		Low	HR = 1.02, P = 0.96195%CI:0.56–1.97	
*miR-150* (High vs Low)	Low	HR = 1.58, P = 0.10495%CI:0.91–2.70	**HR = 1.92,P = 0.03495%CI:1.05**–**3.47**	Low	HR = 1.49, P = 0.17395%CI:0.83–2.56	**HR = 1.94,P = 0.03895%CI:1.04**–**3.56**
*miR-205* (High vs Low)	High	HR = 1.22, P = 0.45995%CI:0.72–2.06		High	HR = 1.39, P = 0.22895%CI:0.81–2.35	
*miR-296* (High vs Low)	High	HR = 1.13, P = 0.83395%CI:0.42–4.65		High	HR = 1.22, P = 0.72595%CI:0.45–5.03	

AA: African-American, ADC: adenocarcinoma, SCC: squamous cell carcinoma, N.S.: not significant, Hazard ratios, 95% confidence intervals and P values were obtained using Cox proportional hazards models for RFS and OS. If significant displayed bold.

**Table 3:** Correlation of clinicopathological and miR factors with clinical outcome in TNM Stage I case (n = 63).

	RFS	Univariate	Multivariable	OS	Univariate	Multivariable
Age (>60 vs≤60)	≤60	HR = 1.01,P = 0.97395%CI:0.41–2.28		>60	HR = 1.19,P = 0.69095%CI:0.52–3.06	
Race (AA vs non-AA)	Non-AA	HR = 1.20,P = 0.70695%CI:0.49–3.63		Non-AA	HR = 1.22,P = 0.68295%CI:0.50–3.67	
Gender (Female vs Male)	Male	HR = 1.09,P = 0.83195%CI:0.49–2.46		Male	HR = 1.08,P = 0.85095%CI:0.50–2.38	
Smoking history (Yes vs No)	Yes	HR = 1.59,P = 0.25095%CI:0.72–3.67		Yes	HR = 1.46,P = 0.33695%CI:0.68–3.27	
Alcohol history (Yes vs No)	Yes	HR = 1.37,P = 0.43695%CI:0.62–3.16		Yes	HR = 1.17,P = 0.69895%CI:0.54–2.58	
Histology (SCC vs ADC)	SCC	HR = 2.13,P = 0.06295%CI:0.96–4.93	**HR** **=** **2.54,P** **=** **0.02695%CI:1.12**–**6.05**	SCC	**HR** **=** **2.28,P** **=** **0.03995%CI:1.04**–**5.23**	**HR** **=** **2.66,P** **=** **0.01695%CI:1.20**–**6.21**
Differentiation (Poor vs Well/Mod)	Poor	HR = 2.50,P = 0.14195%CI:0.71–7.00		Poor	**HR** **=** **3.90, P** **=** **0.02395%CI:1.23**–**10.54**	
*miR-21* (High vs Low)	High	HR = 2.06,P = 0.10495%CI:0.87–5.68		High	HR = 2.19,P = 0.07595%CI:0.93–5.99	
*miR-23b* (High vs Low)	High	HR = 2.00,P = 0.10195%CI:0.87–4.43	**HR** **=** **2.46,P** **=** **0.04195%CI:1.04**–**5.62**	High	**HR** **=** **2.23,P** **=** **0.05195%CI:0.99**–**4.85**	**HR** **=** **2.64,P** **=** **0.02195%CI:1.16**–**5.85**
*miR-31* (High vs Low)	High	HR = 1.19,P = 0.68095%CI:0.53–2.81		High	HR = 1.58,P = 0.26995%CI:0.71–3.87	
*miR-126* (High vs Low)	Low	HR = 1.50,P = 0.32695%CI:0.67–3.54		Low	HR = 1.36,P = 0.43995%CI:0.63–3.12	
*miR-145* (High vs Low)	Low	HR = 1.12,P = 0.80595%CI:0.47–3.08		High	HR = 1.08,P = 0.86495%CI:0.42–2.46	
*miR-150* (High vs Low)	Low	HR = 1.24,P = 0.62695%CI:0.48–2.86		Low	HR = 1.16,P = 0.74095%CI:0.45–2.64	
*miR-205* (High vs Low)	High	HR = 1.75,P = 0.16795%CI:0.79–3.95		High	**HR = 2.20,P = 0.04895%CI:1.01**–**5.03**	
*miR-296* (High vs Low)	High	HR = 1.17,P = 0.87795%CI:0.25–20.85		High	HR = 1.35,P = 0.75795%CI:0.29–24.16	

AA: African-American, ADC: adenocarcinoma, SCC: squamous cell carcinoma, N.S.: not significant, Hazard ratios, 95% confidence intervals and P values were obtained using Cox proportional hazards models for RFS and OS. If significant displayed bold.

**Table 4:** Association of microRNA expression and allelic imbalance.

		ADC (n = 66)		SCC (n = 53)		Total (n = 119)	
microRNA	Locus	Amplification	Deletion	Amplification	Deletion	Amplification	Deletion
*miR-205*	Chr1 q32.2	23/66 (34.8%)	4/66 (6.1%)	6/53 (11.3%)	9/53 (16.7%)	29/119 (24.4%)	13/119 (10.9%)
*miR-296*	Chr20 q13.32	No allelic imbalance				No allelic imbalance	
*miR-21*	Chr17 q23.2	No allelic imbalance				No allelic imbalance	
*miR-23b*	Chr9 q22.32	No allelic imbalance				No allelic imbalance	
*miR-126*	Chr9 q34	4/66 (6.1%)	10/66 (15.2%)	7/53 (13.2%)	12/53 (22.6%)	11/119 (9.2%)	22/119 (18.5%)
*miR-145*	Chr5 q32	No allelic imbalance				No allelic imbalance	
*miR-150*	Chr19 q13.33	8/66 (12.1%)	12/66 (18.2%)	2/53 (3.7%)	7/53 (13.2%)	10/119 (8.4%)	19/119 (16.0%)
*miR-31*	Chr9 p21.3	4/66 (6.1%)	8/66 (12.1%)	2/53 (3.7%)	12/53 (22.6%)	6/119 (5.0%)	20/119 (16.8%)
