# Supplementary material for: An integrated genome-wide approach to discover deregulated microRNAs in non-small cell lung cancer: Clinical significance of miR-23b-3p deregulation
Source: Sci Rep. 2015 Aug 28;5:13236. doi: 10.1038/srep13236 (PMC4551983; doi:10.1038/srep13236)
Supplement: Supplementary Information [file srep13236-s1.doc]

**An integrated genome-wide approach to discover deregulated microRNAs in non-small cell lung cancer: Clinical significance of *miR-23b-3p* deregulation**

**Running title**: *miR-23b-3p* expression in NSCLC

#Shahnaz Begum^1^, #Masamichi Hayashi^2^, Takenori Ogawa^2^, Fayez J. Jabboure^2^, Mariana Brait^2^, Evgeny Izumchenko^2^, Sarit Tabak^5^ , [Steven A. Ahrendt](http://www.sciencedirect.com/science/article/pii/S0046817714001944)^6^, William H. Westra^1^, Wayne Koch^2^, David Sidransky^2^,

*Mohammad O. Hoque ^2,3,4^

^1^Department of Pathology, Johns Hopkins University, Baltimore, Maryland, USA, 21231, ^2^Department of Otolaryngology-Head and Neck Surgery, Johns Hopkins University, ^3^Department of Urology, Johns Hopkins University, ^4^Department of Oncology, Johns Hopkins University, ^5^Rosetta Genomics Ltd. 10 Plaut St., Rehovot, Israel, 76706, ^6^Department of Surgery, Division of Surgical Oncology, University of Pittsburgh Medical Center, Pittsburgh, PA, USA, 15213.

#Contributed equally

***Corresponding author**: Mohammad Obaidul Hoque, DDS, Ph.D.,

Associate Professor of Department of Otolaryngology and Head & Neck Surgery, Urology and Oncology

The Johns Hopkins University School of Medicine

1550 Orleans Street, CRB II, 5M, Baltimore, MD, 21231

Phone 410-502-8778, Fax 410-614-1411, [mhoque1@jhmi.edu](mailto:mhoque1@jhmi.edu)

**Supplementary Table S1**

Clinicopathological information of non-small cell lung cancers for SNP array (n=119)

| **factors** | **ADC (n=66)** | **SCC (n=53)** | **Total (n=119)** |
| --- | --- | --- | --- |
| Age | 64.7 | 67.9 | 66.1 |
| Race (AA/C/Other) | 15/50/1 | 12/40/1 | 27/90/2 |
| Gender (Female/Male) | 36/30 | 18/35 | 54/65 |
| Tumor size (<3cm/<7cm/>7cm) | 35/28/2(1) | 20/26/6(1) | 55/54/8(2) |
| Differentiation (Well/Mod/Poor) | 13/32/15(6) | 3/28/20(2) | 16/60/35(8) |
| TNM stage (I/II/III) | 35/21/10 | 34/12/7 | 69/33/17 |
| Smoking history (Yes/No) | 60/6 | 49/1(3) | 109/7(3) |
| Alcohol history (Yes/No) | 36/23(7) | 33/14(6) | 69/37(13) |

AA: African-American, C: Caucasian, ADC: adenocarcinoma, SCC: squamous cell carcinoma, (number): number of unknown cases

**Supplementary Table S2**

Allelic imbalance regions detected by copy number analysis

ADC (n=66) SCC (n=53)

| **Locus** | **Start** | **End** | **Mb** | **N** | **%** | **N** | % | Associated gene |
| --- | --- | --- | --- | --- | --- | --- | --- | --- |
|  |  |  |  |  |  |  |  |  |
| **1p36.13** | 18754567 | 19486651 | 0.732084 |  |  |  |  | PAX7 |
| **del** |  |  |  | (8/66) | 12.1 | (11/53) | 20.7 | UBR4 |
| **amp** |  |  |  | (8/66) | 12.1 | (4/53) | 7.5 | AKR7A3 |
|  |  |  |  |  |  |  |  |  |
| **1p36.12-1p36.11** | 21527932 | 25275467 | 3.747535 |  |  |  |  | ECE1 |
| **del** |  |  |  | (11/66) | 16.7 | (6/53) | 11.3 | USP48 |
| **amp** |  |  |  |  |  | (9/53) | 16.9 | EPHA8 |
|  |  |  |  |  |  |  |  | C1QA |
|  |  |  |  |  |  |  |  | EPHB2 |
|  |  |  |  |  |  |  |  | IL28RA |
|  |  |  |  |  |  |  |  | CLIC4 |
|  |  |  |  |  |  |  |  | RUNX3 |
|  |  |  |  |  |  |  |  |  |
|  |  |  |  |  |  |  |  |  |
| **1p35.2** | 30665838 | 30800722 | 0.134884 |  |  |  |  | PTPRU |
| **del** |  |  |  | (10/66) | 15.2 | (5/53) | 9.4 |  |
|  |  |  |  |  |  |  |  |  |
| **1p21.3-1p21.2** |  |  |  |  |  |  |  | PTBP2 |
| **del** | 95851805 | 99827387 | 3.975582 |  |  | (12/53) | 22.6 | DPYD |
|  |  |  |  |  |  |  |  | SNX7 |
|  |  |  |  |  |  |  |  | PAP2D |
|  |  |  |  |  |  |  |  | LPPR4 |
|  |  |  |  |  |  |  |  | PALMD |
|  |  |  |  |  |  |  |  |  |
| **1q21.1-1q23.3** | 145155565 | 163480446 | 18.32488 |  |  |  |  | FMO5 |
| **del** |  |  |  | (1/66) | 1.5 | (1/53) | 1.9 | CHD1L |
| **amp** |  |  |  | (15/66) | 22.7 | (9/53) | 16.9 | GJA5 |
|  |  |  |  |  |  |  |  | MTMR11 |
|  |  |  |  |  |  |  |  | SNX27 |
|  |  |  |  |  |  |  |  | S100A10 |
|  |  |  |  |  |  |  |  | FLG2 |
|  |  |  |  |  |  |  |  | FLG |
|  |  |  |  |  |  |  |  | LCE5A |
|  |  |  |  |  |  |  |  | CRNN |
|  |  |  |  |  |  |  |  | LCE3A |
|  |  |  |  |  |  |  |  | LCE1F |
|  |  |  |  |  |  |  |  | S100A9 |
|  |  |  |  |  |  |  |  | ADAR |
|  |  |  |  |  |  |  |  | KCNN3 |
|  |  |  |  |  |  |  |  | NES |
|  |  |  |  |  |  |  |  | FCRL5 |
|  |  |  |  |  |  |  |  | ETV3 |
|  |  |  |  |  |  |  |  | FCRL5 |
|  |  |  |  |  |  |  |  | FCRL4 |
|  |  |  |  |  |  |  |  | KIRREL |
|  |  |  |  |  |  |  |  | OR10R2 |
|  |  |  |  |  |  |  |  | OR6Y1 |
|  |  |  |  |  |  |  |  | SPTA1 |
|  |  |  |  |  |  |  |  | APCS |
|  |  |  |  |  |  |  |  | CRP |
|  |  |  |  |  |  |  |  | SLAMF6 |
|  |  |  |  |  |  |  |  | CD244 |
|  |  |  |  |  |  |  |  | NOS1AP |
|  |  |  |  |  |  |  |  | RGS4 |
|  |  |  |  |  |  |  |  | C1orf110 |
|  |  |  |  |  |  |  |  | PBX1 |
|  |  |  |  |  |  |  |  | LMX1A |
|  |  |  |  |  |  |  |  |  |
| **1q25.1** | 168609996 | 180033060 | 11.42306 |  |  |  |  | SCYL1BP1 |
| **del** |  |  |  |  |  |  |  | BAT2D1 |
| **amp** |  |  |  | (16/66) | 24.2 | (4/53) | 7.5 | PIGC |
|  |  |  |  |  |  |  |  | DNM3 |
|  |  |  |  |  |  |  |  | TNFSF18 |
|  |  |  |  |  |  |  |  | SLC9A11 |
|  |  |  |  |  |  |  |  | RC3H1 |
|  |  |  |  |  |  |  |  | RABGAP1L |
|  |  |  |  |  |  |  |  | TNR |
|  |  |  |  |  |  |  |  | PAPPA2 |
|  |  |  |  |  |  |  |  | FAM5B |
|  |  |  |  |  |  |  |  | ABL2 |
|  |  |  |  |  |  |  |  | NPHS2 |
|  |  |  |  |  |  |  |  | ACBD6 |
|  |  |  |  |  |  |  |  | CACNA1E |
|  |  |  |  |  |  |  |  |  |
| **1q25.3-1q31.1** | 183402368 | 194941337 | 11.53897 |  |  |  |  | C1orf26 |
| **del** |  |  |  | (4/66) | 6.1 | (7/53) | 13.2 | IVNS1ABP |
| **amp** |  |  |  | (21/66) | 31.8 | (4/53) | 7.5 | HMCN1 |
|  |  |  |  |  |  |  |  | TPR |
|  |  |  |  |  |  |  |  | FAM5C |
|  |  |  |  |  |  |  |  | RGS18 |
|  |  |  |  |  |  |  |  | UCHL5 |
|  |  |  |  |  |  |  |  | CDC73 |
|  |  |  |  |  |  |  |  | KCNT2 |
|  |  |  |  |  |  |  |  | CFH |
|  |  |  |  |  |  |  |  |  |
| **1q32.2-q41** | 209007493 | 216813781 | 7.806288 |  |  |  |  | KCNH1 |
| **del** |  |  |  | (4/66) | 6.1 | (9/53) | 16.7 | RD3 |
| **amp** |  |  |  | (23/66) | 34.8 | (6/53) | 11.3 | DTL |
|  |  |  |  |  |  |  |  | PPP2R5A |
|  |  |  |  |  |  |  |  | FLVCR1 |
|  |  |  |  |  |  |  |  | KCNK2 |
|  |  |  |  |  |  |  |  | CENPF |
|  |  |  |  |  |  |  |  | USH2A |
|  |  |  |  |  |  |  |  | ESRRG |
|  |  |  |  |  |  |  |  | SPATA17 |
|  |  |  |  |  |  |  |  | LYPLAL1 |
|  |  |  |  |  |  |  |  |  |
| **2p22.1-2p21** | 40240967 | 40956727 | 0.71576 |  |  |  |  | SLC8A1 |
| **del** |  |  |  | (17/66) | 25.8 | (8/53) | 15.1 |  |
| **amp** |  |  |  | (12/66) | 18.2 | (7/53) | 13.2 |  |
|  |  |  |  |  |  |  |  |  |
| **2p14-p13.3** | 66497277 | 71453862 | 4.956585 |  |  |  |  | MEIS1 |
| **del** |  |  |  | (6/66) | 9.1 | (10/53) | 18.9 | ETAA1 |
| **amp** |  |  |  | (4/66) | 6.1 | (2/53) | 3.7 | C1D |
|  |  |  |  |  |  |  |  | ARHGAP25 |
|  |  |  |  |  |  |  |  | AAK1 |
|  |  |  |  |  |  |  |  | TGFA |
|  |  |  |  |  |  |  |  | MPHOSPH10 |
|  |  |  |  |  |  |  |  | PAIP2B |
|  |  |  |  |  |  |  |  | ZNF638 |
|  |  |  |  |  |  |  |  |  |
| **2p12** | 79079053 | 81449210 | 2.370157 |  |  |  |  | REG3G |
| **del** |  |  |  | (3/66) | 4.5 | (8/53) | 15.1 | CTNNA2 |
| **amp** |  |  |  | (3/66) | 4.5 | (4/53) | 7.4 |  |
|  |  |  |  |  |  |  |  |  |
| **2q22.3-q23.1** | 146607543 | 148735075 | 2.127532 |  |  |  |  | LOC728773 |
| **del** |  |  |  | (9/66) | 13.6 | (10/53) | 18.8 | ACVR2A |
| **amp** |  |  |  | (7/66) | 10.6 | (6/53) | 11.3 | MBD5 |
|  |  |  |  |  |  |  |  |  |
| **2q36.3** | 229212592 | 230442325 | 1.229733 |  |  |  |  | SPHKAP |
| **del** |  |  |  | (5/66) | 7.6 | (10/53) | 18.9 | PID1 |
| **amp** |  |  |  | (4/66) | 6.1 | (2/53) | 3.7 |  |
|  |  |  |  |  |  |  |  |  |
| **2q34** | 209269970 | 214915361 | 5.645391 |  |  |  |  | PTH2R |
| **del** |  |  |  | (6/66) | 9.1 | (13/53) | 24.6 | MAP2 |
| **amp** |  |  |  | (6/66) | 9.1 | (1/53) | 1.8 | MYL1 |
|  |  |  |  |  |  |  |  | LANCL1 |
|  |  |  |  |  |  |  |  | ERBB4 |
|  |  |  |  |  |  |  |  | IKZF2 |
|  |  |  |  |  |  |  |  | SPAG16 |
|  |  |  |  |  |  |  |  |  |
| **2q24.2** | 163139654 | 165402982 | 2.263328 |  |  |  |  | KCNH7 |
| **del** |  |  |  | (9/66) | 13.6 | (9/53) | 16.9 | FIGN |
| **amp** |  |  |  | (4/66) | 6.1 | (3/53) | 5.6 | COBLL1 |
|  |  |  |  |  |  |  |  | DNER |
|  |  |  |  |  |  |  |  | TRIP12 |
|  |  |  |  |  |  |  |  | SP110 |
|  |  |  |  |  |  |  |  |  |
| **2q37.1-q37.3** | 235044405 | 237342667 | 2.298262 |  |  |  |  | SH3BP4 |
| **del** |  |  |  | (13/66) | 19.7 | (6/53) | 11.3 | CENTG2 |
| **amp** |  |  |  | (5/66) | 7.6 | (3/53) | 5.6 |  |
|  |  |  |  |  |  |  |  |  |
| **2q37.3** | 237395901 | 240571495 | 3.175594 |  |  |  |  | CXCR7 |
| **del** |  |  |  | (5/66) | 7.6 | (10/53) | 18.8 | COL6A3 |
| **amp** |  |  |  | (5/66) | 7.6 | (4/53) | 7.4 | LRRFIP1 |
|  |  |  |  |  |  |  |  | ASB1 |
|  |  |  |  |  |  |  |  | HDAC4 |
|  |  |  |  |  |  |  |  | NDUFA10 |
|  |  |  |  |  |  |  |  |  |
| **3q23-q24** | 142087750 | 146670125 | 4.582375 |  |  |  |  | TRIM42 |
| **del** |  |  |  | (3/66) | 4.5 | (3/53) | 5.6 | ZBTB38 |
| **amp** |  |  |  | (8/66) | 12.1 | (30/53) | 56.6 | GRK7 |
|  |  |  |  |  |  |  |  | TRPC1 |
|  |  |  |  |  |  |  |  | SR140 |
|  |  |  |  |  |  |  |  | CHST2 |
|  |  |  |  |  |  |  |  | SLC9A9 |
|  |  |  |  |  |  |  |  | C3orf58 |
|  |  |  |  |  |  |  |  |  |
| **3q24-q25.32** | 149081888 | 159848232 | 10.76634 |  |  |  |  | ZIC1 |
| **del** |  |  |  | (4/66) | 6.1 | (3/53) | 5.6 | AGTR1 |
| **amp** |  |  |  | (7/66) | 10.6 | (25/53) | 47.2 | CPA3 |
|  |  |  |  |  |  |  |  | WWTR1 |
|  |  |  |  |  |  |  |  | COMMD2 |
|  |  |  |  |  |  |  |  | RNF13 |
|  |  |  |  |  |  |  |  | TSC22D2 |
|  |  |  |  |  |  |  |  | MED12L |
|  |  |  |  |  |  |  |  | AADACL2 |
|  |  |  |  |  |  |  |  | MBNL1 |
|  |  |  |  |  |  |  |  | P2RY1 |
|  |  |  |  |  |  |  |  | KCNAB1 |
|  |  |  |  |  |  |  |  | TIPARP |
|  |  |  |  |  |  |  |  | VEPH1 |
|  |  |  |  |  |  |  |  | RSRC1 |
|  |  |  |  |  |  |  |  |  |
| **3p26.3-26.2** | 540961 | 4001680 | 3.460719 |  |  |  |  | CNTN6 |
| **del** |  |  |  | (10/66) | 15.2 | (17/53) | 32.1 | CNTN4 |
| **amp** |  |  |  | (9/66) | 13.6 | (3/53) | 5.6 | SETMAR |
|  |  |  |  |  |  |  |  | TRNT1 |
|  |  |  |  |  |  |  |  | SUMF1 |
|  |  |  |  |  |  |  |  | CRBN |
|  |  |  |  |  |  |  |  | TRNT1 |
|  |  |  |  |  |  |  |  |  |
| **3p26.1** |  |  |  |  |  |  |  | GRM7 |
| **del** |  |  |  | (10/66) | 15.2 | (13/53) | 24.6 |  |
| **amp** |  |  |  | (5/66) | 7.6 | (4/53) | 7.5 |  |
|  |  |  |  |  |  |  |  |  |
| **3p24.1** | 27045335 | 30431532 | 3.386197 |  |  |  |  | NEK10 |
| **del** |  |  |  | (15/66) | 22.7 | (14/53) | 26.4 | EOMES |
| **amp** |  |  |  | (5/66) | 7.6 | (3/53) | 5.6 | CMC1 |
|  |  |  |  |  |  |  |  | RBMS3 |
|  |  |  |  |  |  |  |  | TGFBR2 |
|  |  |  |  |  |  |  |  |  |
| **3p14.3** | 54903802 | 55927455 | 1.023653 |  |  |  |  | LRTM1 |
| **del** |  |  |  | (8/66) | 12.1 | (11/53) | 20.7 | WNT5A |
| **amp** |  |  |  | (3/66) | 4.5 | (1/53) | 1.8 | CACNA2D3 |
|  |  |  |  |  |  |  |  |  |
| **3p14.2** | 59585967 | 62679690 | 3.093723 |  |  |  |  | WNT5A |
| **del** |  |  |  | (7/66) | 10.6 | (14/53) | 26.4 | FHIT |
| **amp** |  |  |  | (6/66) | 9.1 |  |  | PTPRG |
|  |  |  |  |  |  |  |  | ID2 |
|  |  |  |  |  |  |  |  | CADPS |
|  |  |  |  |  |  |  |  |  |
| **3p12.3-12.1** | 78990861 | 84484209 | 5.493348 |  |  |  |  | ROBO1 |
| **del** |  |  |  | (14/66) | 21.2 | (12/53) | 22.6 | GBE1 |
| **amp** |  |  |  | (3/66) | 4.5 | (1/53) | 1.8 | CADM2 |
|  |  |  |  |  |  |  |  |  |
| **3q22.1-22.3** | 135082860 | 139139288 | 4.056428 |  |  |  |  | RAB6A |
| **del** |  |  |  | (5/66) | 7.6 | (4/53) | 7.5 | KY |
| **amp** |  |  |  | (9/66) | 13.6 | (15/53) | 28.3 | EPHB1 |
|  |  |  |  |  |  |  |  | IL20RB |
|  |  |  |  |  |  |  |  | CLDN18 |
|  |  |  |  |  |  |  |  | SOX14 |
|  |  |  |  |  |  |  |  | CLDN18 |
|  |  |  |  |  |  |  |  |  |
| **3q26.1-3q29** | 164484723 | 199364601 | 34.87988 |  |  |  |  | SI |
| **del** |  |  |  | (2/66) | 3.0 | (3/53) | 5.6 | BCHE |
| **amp** |  |  |  | (6/66) | 9.1 | (14/53) | 26.4 | SERPINI1 |
|  |  |  |  |  |  |  |  | GOLIM4 |
|  |  |  |  |  |  |  |  | EVI1 |
|  |  |  |  |  |  |  |  | MDS1 |
|  |  |  |  |  |  |  |  | LRRC31 |
|  |  |  |  |  |  |  |  | SAMD7 |
|  |  |  |  |  |  |  |  | TNIK |
|  |  |  |  |  |  |  |  | PLD1 |
|  |  |  |  |  |  |  |  | FNDC3B |
|  |  |  |  |  |  |  |  | SPATA16 |
|  |  |  |  |  |  |  |  | NLGN1 |
|  |  |  |  |  |  |  |  | NAALADL2 |
|  |  |  |  |  |  |  |  | TBL1XR1 |
|  |  |  |  |  |  |  |  | KCNMB2 |
|  |  |  |  |  |  |  |  | PIK3CA |
|  |  |  |  |  |  |  |  | ACTL6A |
|  |  |  |  |  |  |  |  | PEX5L |
|  |  |  |  |  |  |  |  | FXR1 |
|  |  |  |  |  |  |  |  | SOX2 |
|  |  |  |  |  |  |  |  | ATP11B |
|  |  |  |  |  |  |  |  | LAMP3 |
|  |  |  |  |  |  |  |  | MCF2L2 |
|  |  |  |  |  |  |  |  | YEATS2 |
|  |  |  |  |  |  |  |  | PARL |
|  |  |  |  |  |  |  |  | ABCC5 |
|  |  |  |  |  |  |  |  | MAGEF1 |
|  |  |  |  |  |  |  |  | VPS8 |
|  |  |  |  |  |  |  |  | EHHADH |
|  |  |  |  |  |  |  |  | SST |
|  |  |  |  |  |  |  |  | RTP2 |
|  |  |  |  |  |  |  |  | BCL6 |
|  |  |  |  |  |  |  |  | LPP |
|  |  |  |  |  |  |  |  | TPRG1 |
|  |  |  |  |  |  |  |  | LEPREL1 |
|  |  |  |  |  |  |  |  | CLDN1 |
|  |  |  |  |  |  |  |  | OSTN |
|  |  |  |  |  |  |  |  | CCDC50 |
|  |  |  |  |  |  |  |  | UTS2D |
|  |  |  |  |  |  |  |  | FGF12 |
|  |  |  |  |  |  |  |  | C3orf59 |
|  |  |  |  |  |  |  |  | HRASLS |
|  |  |  |  |  |  |  |  | ATP13A5 |
|  |  |  |  |  |  |  |  | HES1 |
|  |  |  |  |  |  |  |  | LSG1 |
|  |  |  |  |  |  |  |  | C3orf21 |
|  |  |  |  |  |  |  |  | WDR53 |
|  |  |  |  |  |  |  |  | DLG1 |
|  |  |  |  |  |  |  |  |  |
| **4p16.1-p15.33** | 9713768 | 12551237 | 2.837469 |  |  |  |  | WDR1 |
| **del** |  |  |  | (7/66) | 10.6 | (16/53) | 30.2 | MIST |
| **amp** |  |  |  | (6/66) | 9.1 |  |  | HS3ST1 |
|  |  |  |  |  |  |  |  | HSP90AB2P |
|  |  |  |  |  |  |  |  |  |
| **4p14-p13** | 36607819 | 45284259 | 8.67644 |  |  |  |  | FLJ16686 |
| **del** |  |  |  | (5/66) | 7.6 | (12/53) | 22.6 | KIAA1239 |
| **amp** |  |  |  | (4/66) | 6.1 | (3/53) | 5.6 | TBC1D1 |
|  |  |  |  |  |  |  |  | FAM114A1 |
|  |  |  |  |  |  |  |  | KLHL5 |
|  |  |  |  |  |  |  |  | RBM47 |
|  |  |  |  |  |  |  |  | APBB2 |
|  |  |  |  |  |  |  |  | UCHL1 |
|  |  |  |  |  |  |  |  | ATP8A1 |
|  |  |  |  |  |  |  |  | YIPF7 |
|  |  |  |  |  |  |  |  | GNPDA2 |
|  |  |  |  |  |  |  |  |  |
| **4q13.1** | 60846313 | 68541139 | 7.694826 |  |  |  |  | LPHN3 |
| **del** |  |  |  | (10/66) | 15.2 | (12/53) | 22.6 | SRD5A2L2 |
| **amp** |  |  |  | (7/66) | 10.6 | (5/53) | 9.4 | EPHA5 |
|  |  |  |  |  |  |  |  | TMPRSS11A |
|  |  |  |  |  |  |  |  |  |
| **4q21.21** | 82233908 | 83130142 | 0.896234 |  |  |  |  | RASGEF1B |
| **del** |  |  |  | (6/66) | 9.1 | (17/53) | 32.0 |  |
| **amp** |  |  |  | (3/66) | 4.5 | (3/53) | 5.6 |  |
|  |  |  |  |  |  |  |  |  |
| **4q22.1** | 91190768 | 93219460 | 2.028692 |  |  |  |  | MGC48628 |
| **del** |  |  |  | (4/66) | 6.1 | (13/53) | 24.5 | GRID2 |
| **amp** |  |  |  | (5/66) | 7.6 | (4/53) | 7.5 |  |
|  |  |  |  |  |  |  |  |  |
| **4q31.22** | 141771249 | 144901159 | 3.12991 |  |  |  |  | TBC1D9 |
| **del** |  |  |  | (14/66) | 21.2 | (11/53) | 20.7 | RNF150 |
| **amp** |  |  |  | (3/66) | 4.5 | (1/53) | 1.8 | INPP4B |
|  |  |  |  |  |  |  |  | IL15 |
|  |  |  |  |  |  |  |  | INPP4B |
|  |  |  |  |  |  |  |  | USP38 |
|  |  |  |  |  |  |  |  | GYPE |
|  |  |  |  |  |  |  |  |  |
| **4q28.3** | 131476725 | 139124422 | 7.647697 |  |  |  |  | PCDH10 |
| **del** |  |  |  | (6/66) | 9.1 | (11/53) | 20.7 | PCDH18 |
| **amp** |  |  |  | (4/66) | 6.1 | (1/53) | 1.8 | SLC7A11 |
|  |  |  |  |  |  |  |  |  |
| **4q34.1-34.2** | 172370512 | 177472089 | 5.101577 |  |  |  |  | GALNT17 |
| **del** |  |  |  | (9/66) | 13.6 | (12/53) | 22.6 | SCRG1 |
| **amp** |  |  |  | (3/66) | 4.5 | (2/53) | 3.7 | HAND2 |
|  |  |  |  |  |  |  |  | GLRA3 |
|  |  |  |  |  |  |  |  | ADAM29 |
|  |  |  |  |  |  |  |  | GPM6A |
|  |  |  |  |  |  |  |  |  |
| **4q35.1-q35.2** | 186427288 | 189532033 | 3.104745 |  |  |  |  | SNX25 |
| **del** |  |  |  | (10/66) | 15.2 | (18/53) | 33.9 | SORBS2 |
| **amp** |  |  |  | (5/66) | 7.6 | (1/53) | 3.7 | KLKB1 |
|  |  |  |  |  |  |  |  | F11 |
|  |  |  |  |  |  |  |  | FAT |
|  |  |  |  |  |  |  |  | TRIML2 |
|  |  |  |  |  |  |  |  | TRIML1 |
|  |  |  |  |  |  |  |  |  |
| **5q21.2-q21.3** | 103668828 | 106284527 | 2.615699 |  |  |  |  | EFNA5 |
| **del** |  |  |  | (15/66) | 22.7 | (17/53) | 32.0 | FBXL17 |
| **amp** |  |  |  | (11/66) | 16.7 | (4/53) | 7.5 |  |
|  |  |  |  |  |  |  |  |  |
| **5q11.1-11.2** | 49596616 | 52787113 | 3.190497 |  |  |  |  | ISL1 |
| **del** |  |  |  | (14/66) | 21.2 | (11/53) | 20.7 | MOCS2 |
| **amp** |  |  |  | (7/66) | 10.6 | (2/53) | 3.7 | FST |
|  |  |  |  |  |  |  |  | ARL15 |
|  |  |  |  |  |  |  |  |  |
| **5p13.1-p12** | 41338684 | 44478700 | 3.140016 |  |  |  |  | C7 |
| **del** |  |  |  | (6/66) | 9.1 | (6/53) | 11.3 | FLJ40243 |
| **amp** |  |  |  | (17/66) | 25.8 | (16/53) | 30.1 | PLCXD3 |
|  |  |  |  |  |  |  |  | OXCT1 |
|  |  |  |  |  |  |  |  | PLCXD3 |
|  |  |  |  |  |  |  |  | GHR |
|  |  |  |  |  |  |  |  | FGF10 |
|  |  |  |  |  |  |  |  |  |
| **5q14.1** | 77952104 | 79989532 | 2.037428 |  |  |  |  | ARSB |
| **del** |  |  |  | (13/66) | 19.7 | (12/53) | 22.6 | CMYA5 |
| **amp** |  |  |  | (5/66) | 7.6 | (4/53) | 7.5 | THBS4 |
|  |  |  |  |  |  |  |  | SERINC5 |
|  |  |  |  |  |  |  |  | ZFYVE16 |
|  |  |  |  |  |  |  |  | FAM151B |
|  |  |  |  |  |  |  |  | MSH3 |
|  |  |  |  |  |  |  |  |  |
| **5p14.3-5p13.3** | 18750887 | 31683514 | 12.93263 |  |  |  |  | CDH18 |
| **del** |  |  |  | (3/66) | 4.5 | (4/53) | 7.5 | CDH12 |
| **amp** |  |  |  | (16/66) | 24.2 | (14/53) | 26.4 | PRDM9 |
|  |  |  |  |  |  |  |  | CDH9 |
|  |  |  |  |  |  |  |  | CDH6 |
|  |  |  |  |  |  |  |  | RNASEN |
|  |  |  |  |  |  |  |  | PDZD2 |
|  |  |  |  |  |  |  |  |  |
| **5p15.32-p15.31** | 5020646 | 6844552 | 1.823906 |  |  |  |  | IRX1 |
| **del** |  |  |  | (9/53) | 17.0 | (6/53) | 11.3 | ADAMTS16 |
| **amp** |  |  |  | (12/66) | 18.2 | (17/53) | 32.0 | FLJ33360 |
|  |  |  |  |  |  |  |  | KIAA0947 |
|  |  |  |  |  |  |  |  |  |
| **6q21-q22.1** | 112348847 | 115895494 | 3.546647 |  |  |  |  | FYN |
| **del** |  |  |  | (14/66) | 21.2 | (8/53) | 15.1 | LAMA4 |
| **amp** |  |  |  | (4/66) | 6.1 | (1/53) | 1.8 | HDAC2 |
|  |  |  |  |  |  |  |  | FRK |
|  |  |  |  |  |  |  |  |  |
| **6q25.1** | 151295075 | 152489412 | 1.194337 |  |  |  |  | C6orf97 |
| **del** |  |  |  | (13/66) | 19.7 | (8/53) | 15.1 | ESR1 |
| **amp** |  |  |  | (4/66) | 6.1 | (3/53) | 5.6 | SYNE1 |
|  |  |  |  |  |  |  |  |  |
| **6p25.1-p24.3** | 4865084 | 8426832 | 3.561748 |  |  |  |  | CDYL |
| **del** |  |  |  | (2/66) | 3.0 | (6/53) | 11.3 | FARS2 |
| **amp** |  |  |  | (20/66) | 30.3 | (6/53) | 11.3 | F13A1 |
|  |  |  |  |  |  |  |  | LY86 |
|  |  |  |  |  |  |  |  | RREB1 |
|  |  |  |  |  |  |  |  | RIOK1 |
|  |  |  |  |  |  |  |  | C6orf151 |
|  |  |  |  |  |  |  |  | TXNDC5 |
|  |  |  |  |  |  |  |  | EEF1E1 |
|  |  |  |  |  |  |  |  | OFCC1 |
|  |  |  |  |  |  |  |  | SLC35B3 |
|  |  |  |  |  |  |  |  |  |
| **6p23-p22.3** | 14676648 | 16186293 | 1.509645 |  |  |  |  | RNF182 |
| **del** |  |  |  | (4/66) | 6.1 | (8/53) | 15.1 | DTNBP1 |
| **amp** |  |  |  | (14/66) | 21.2 | (3/53) | 5.6 | ATXN1 |
|  |  |  |  |  |  |  |  |  |
| **6q15** | 88606514 | 92098763 | 3.492249 |  |  |  |  | SRrp35 |
| **del** |  |  |  | (12/66) | 18.2 | (7/53) | 13.2 | MAP3K7 |
| **amp** |  |  |  | (5/66) | 7.6 | (3/53) | 5.6 |  |
|  |  |  |  |  |  |  |  |  |
|  |  |  |  |  |  |  |  |  |
| **6q26** | 161008908 | 161807615 | 0.798707 |  |  |  |  | PLG |
| **del** |  |  |  | (12/66) | 18.2 | (7/53) | 13.2 | MAP3K4 |
| **amp** |  |  |  | (2/66) | 3.0 | (3/53) | 5.6 | AGPAT4 |
|  |  |  |  |  |  |  |  | PARK2 |
|  |  |  |  |  |  |  |  |  |
| **7q11-q21.11** | 67797528 | 78159892 | 10.36236 |  |  |  |  | AUTS2 |
| **del** |  |  |  | (5/66) | 7.6 | (12/53) | 22.6 | WBSCR17 |
| **amp** |  |  |  | (5/66) | 7.6 | (2/53) | 3.7 | CALN1 |
|  |  |  |  |  |  |  |  | MAGI2 |
|  |  |  |  |  |  |  |  |  |
| **7p21.2-p21.1** | 15009132 | 17527274 | 2.518142 |  |  |  |  | TMEM195 |
| **del** |  |  |  | (2/66) | 3.0 | (8/53) | 15.1 | DGKB |
| **amp** |  |  |  | (17/66) | 25.8 | (7/53) | 13.2 | TSPAN13 |
|  |  |  |  |  |  |  |  | AGR3 |
|  |  |  |  |  |  |  |  | SNX13 |
|  |  |  |  |  |  |  |  |  |
| **8p23.2** | 1130901 | 5255776 | 4.124875 |  |  |  |  | DLGAP2 |
| **del** |  |  |  | (12/66) | 18.2 | (24/53) | 45.3 | MYOM2 |
| **amp** |  |  |  | (8/66) | 12.1 | (5/53) | 9.4 | CSMD1 |
|  |  |  |  |  |  |  |  |  |
| **8p22** | 14005910 | 16867896 | 2.861986 |  |  |  |  | SGCZ |
| **del** |  |  |  | (11/66) | 16.7 | (18/53) | 34.0 | TUSC3 |
| **amp** |  |  |  | (6/66) | 9.1 | (3/53) | 5.7 | FGF20 |
|  |  |  |  |  |  |  |  | MSR1 |
|  |  |  |  |  |  |  |  |  |
| **8p11.21** | 40156816 | 40607130 | 0.450314 |  |  |  |  | ZMAT4 |
| **del** |  |  |  | (5/66) | 7.6 | (8/53) | 15.1 | C8orf4 |
| **amp** |  |  |  | (7/66) | 10.6 | (8/53) | 15.1 |  |
|  |  |  |  |  |  |  |  |  |
| **8q11.23** | 53481177 | 55372771 | 1.891594 |  |  |  |  | FAM150A |
| **del** |  |  |  | (3/66) | 4.5 | (7/53) | 13.2 | NPBWR1 |
| **amp** |  |  |  | (14/66) | 21.2 | (5/53) | 9.4 | OPRK1 |
|  |  |  |  |  |  |  |  | NPBWR1 |
|  |  |  |  |  |  |  |  | ATP6V1H |
|  |  |  |  |  |  |  |  |  |
| **8q12.2-12.3** | 62166007 | 65530781 | 3.364774 |  |  |  |  | ASPH |
| **del** |  |  |  | (5/66) | 7.6 | (8/53) | 15.1 | NKAIN3 |
| **amp** |  |  |  | (14/66) | 21.2 | (11/53) | 20.7 |  |
|  |  |  |  |  |  |  |  |  |
| **8q13.1-13.2** | 67387124 | 72728798 | 5.341674 |  |  |  |  | CRH |
| **del** |  |  |  | (5/66) | 7.6 | (8/53) | 15.1 | CPA6 |
| **amp** |  |  |  | (18/66) | 27.3 | (10/53) | 18.8 | C8orf34 |
|  |  |  |  |  |  |  |  | SULF1 |
|  |  |  |  |  |  |  |  | SLCO5A1 |
|  |  |  |  |  |  |  |  | PRDM14 |
|  |  |  |  |  |  |  |  | NCOA2 |
|  |  |  |  |  |  |  |  | EYA1 |
|  |  |  |  |  |  |  |  |  |
| **8q21.12-21.2** | 78714257 | 85990298 | 7.276041 |  |  |  |  | PXMP3 |
| **del** |  |  |  | (3/66) | 4.5 | (9/53) | 16.9 | FAM164A |
| **amp** |  |  |  | (19/66) | 28.8 | (12/53) | 22.6 | STMN2 |
|  |  |  |  |  |  |  |  | TPD52 |
|  |  |  |  |  |  |  |  | FABP5 |
|  |  |  |  |  |  |  |  | SNX16 |
|  |  |  |  |  |  |  |  | RALYL |
|  |  |  |  |  |  |  |  |  |
| **8q21.3-22.3** | 90614908 | 105778412 | 15.1635 |  |  |  |  | RIPK2 |
| **del** |  |  |  | (3/66) | 4.5 | (5/53) | 9.4 | DECR1 |
| **amp** |  |  |  | (20/66) | 30.3 | (13/53) | 24.5 | TMEM64 |
|  |  |  |  |  |  |  |  | NECAB1 |
|  |  |  |  |  |  |  |  | RUNX1T1 |
|  |  |  |  |  |  |  |  | C8orf83 |
|  |  |  |  |  |  |  |  | PPM2C |
|  |  |  |  |  |  |  |  | CDH17 |
|  |  |  |  |  |  |  |  | GEM |
|  |  |  |  |  |  |  |  | PGCP |
|  |  |  |  |  |  |  |  | MTDH |
|  |  |  |  |  |  |  |  | VPS13B |
|  |  |  |  |  |  |  |  | RGS22 |
|  |  |  |  |  |  |  |  | ANKRD46 |
|  |  |  |  |  |  |  |  | RNF19A |
|  |  |  |  |  |  |  |  | SNX31 |
|  |  |  |  |  |  |  |  | YWHAZ |
|  |  |  |  |  |  |  |  | NCALD |
|  |  |  |  |  |  |  |  | RIMS2 |
|  |  |  |  |  |  |  |  | DPYS |
|  |  |  |  |  |  |  |  | ZFPM2 |
|  |  |  |  |  |  |  |  |  |
| **8q23.1-q23.3** | 109023057 | 114088316 | 5.065259 |  |  |  |  | RSPO2 |
| **del** |  |  |  | (4/66) | 6.1 | (7/53) | 13.2 | TMEM74 |
| **amp** |  |  |  | (16/66) | 24.2 | (15/53) | 28.3 | PKHD1L1 |
|  |  |  |  |  |  |  |  | GOLSYN |
|  |  |  |  |  |  |  |  | KCNV1 |
|  |  |  |  |  |  |  |  | CSMD3 |
|  |  |  |  |  |  |  |  |  |
| **8q24.21-q24.23** | 131094216 | 137886588 | 6.792372 |  |  |  |  | DDEF1 |
| **del** |  |  |  | (5/66) | 7.6 | (10/53) | 18.9 | ADCY8 |
| **amp** |  |  |  | (19/66) | 28.8 | (7/53) | 13.2 | EFR3A |
|  |  |  |  |  |  |  |  | ZFAT |
|  |  |  |  |  |  |  |  | KHDRBS3 |
|  |  |  |  |  |  |  |  |  |
| **8q24.11-24.21** | 117985196 | 129018625 | 11.03343 |  |  |  |  | RAD21 |
| **del** |  |  |  | (3/66) | 4.5 | (5/53) | 9.4 | LOC441376 |
| **amp** |  |  |  | (17/66) | 25.8 | (11/53) | 20.7 | SLC30A8 |
|  |  |  |  |  |  |  |  | MED30 |
|  |  |  |  |  |  |  |  | SAMD12 |
|  |  |  |  |  |  |  |  | TNFRSF11B |
|  |  |  |  |  |  |  |  | COLEC10 |
|  |  |  |  |  |  |  |  | ENPP2 |
|  |  |  |  |  |  |  |  | NOV |
|  |  |  |  |  |  |  |  | ENPP2 |
|  |  |  |  |  |  |  |  | SNTB1 |
|  |  |  |  |  |  |  |  | HAS2 |
|  |  |  |  |  |  |  |  | SNTB1 |
|  |  |  |  |  |  |  |  | WDR67 |
|  |  |  |  |  |  |  |  | ANXA13 |
|  |  |  |  |  |  |  |  | ZNF572 |
|  |  |  |  |  |  |  |  | MTSS1 |
|  |  |  |  |  |  |  |  | MYC |
|  |  |  |  |  |  |  |  | TMEM75 |
|  |  |  |  |  |  |  |  |  |
| **9p24.3** | 239391 | 4094571 | 3.85518 |  |  |  |  | DOCK8 |
| **del** |  |  |  | (12/66) | 18.2 | (13/53) | 24.5 | KANK1 |
| **amp** |  |  |  | (3/66) | 4.5 | (4/53) | 7.5 | SMARCA2 |
|  |  |  |  |  |  |  |  | VLDLR |
|  |  |  |  |  |  |  |  | RFX3 |
|  |  |  |  |  |  |  |  | GPS2 |
|  |  |  |  |  |  |  |  | GLIS3 |
|  |  |  |  |  |  |  |  |  |
| **9p24.1-24.3** | 8149670 | 10004143 | 1.854473 |  |  |  |  | PTPRD |
| **del** |  |  |  | (10/66) | 15.2 | (11/53) | 20.8 |  |
| **amp** |  |  |  | (4/66) | 6.1 | (3/53) | 5.6 |  |
|  |  |  |  |  |  |  |  |  |
| **9p21.3** | 21283631 | 24576464 | 3.292833 |  |  |  |  | IFNA8 |
| **del** |  |  |  | (14/66) | 21.2 | (16/53) | 30.2 | CDKN2B |
| **amp** |  |  |  | (3/66) | 4.5 | (2/53) | 3.7 | MTAP |
|  |  |  |  |  |  |  |  | NSG-x |
|  |  |  |  |  |  |  |  | ELAVL2 |
|  |  |  |  |  |  |  |  |  |
| **9p21.2-21.1** | 28026991 | 32506864 | 4.479873 |  |  |  |  | LINGO2 |
| **del** |  |  |  | (13/66) | 19.7 | (14/53) | 26.4 |  |
| **amp** |  |  |  | (5/66) | 7.6 | (3/53) | 5.6 |  |
|  |  |  |  |  |  |  |  |  |
| **9p13.1** | 38017644 | 38288618 | 0.270974 |  |  |  |  | ALDH1B1 |
| **del** |  |  |  | (9/66) | 13.6 | (10/53) | 18.9 |  |
| **amp** |  |  |  | (9/66) | 13.6 | (7/53) | 13.2 |  |
|  |  |  |  |  |  |  |  |  |
| **9p24.1** | 6108911 | 7703572 | 1.594661 |  |  |  |  | IL33 |
| **del** |  |  |  | (21/66) | 31.8 | (14/53) | 26.4 | RANBP6 |
| **amp** |  |  |  | (7/66) | 10.6 | (3/53) | 5.6 | UHRF2 |
|  |  |  |  |  |  |  |  | TPD52L3 |
|  |  |  |  |  |  |  |  | GLDC |
|  |  |  |  |  |  |  |  | JMJD2C |
|  |  |  |  |  |  |  |  |  |
| **9q21.2-21.31** | 79894551 | 82033313 | 2.138762 |  |  |  |  | GNAQ |
| **del** |  |  |  | (8/66) | 12.1 | (12/53) | 22.6 | PSAT1 |
| **amp** |  |  |  | (4/66) | 6.1 | (2/53) | 3.7 | CHCHD9 |
|  |  |  |  |  |  |  |  | TLE4 |
|  |  |  |  |  |  |  |  |  |
| **9q31.1** | 103049402 | 105906927 | 2.857525 |  |  |  |  | RP11-35N6.1 |
| **del** |  |  |  | (8/66) | 12.1 | (12/53) | 22.6 | GRIN3A |
| **amp** |  |  |  | (7/66) | 10.6 | (2/53) | 3.7 | CYLC2 |
|  |  |  |  |  |  |  |  | SMC2 |
|  |  |  |  |  |  |  |  |  |
| **9q33.1** | 116863722 | 121284576 | 4.420854 |  |  |  |  | ASTN2 |
| **del** |  |  |  | (16/66) | 24.2 | (11/53) | 20.8 | TLR4 |
| **amp** |  |  |  | (6/66) | 9.1 | (5/53) | 9.4 | DBC1 |
|  |  |  |  |  |  |  |  |  |
|  |  |  |  |  |  |  |  |  |
| **9q33.2-q34.13** | 125537083 | 134554767 | 9.017684 |  |  |  |  | DENND1A |
| **del** |  |  |  | (10/66) | 15.2 | (12/53) | 22.6 | MAPKAP1 |
| **amp** |  |  |  | (4/66) | 6.1 | (7/53) | 13.2 | FAM125B |
|  |  |  |  |  |  |  |  | FPGS |
|  |  |  |  |  |  |  |  | CRAT |
|  |  |  |  |  |  |  |  | LAMC3 |
|  |  |  |  |  |  |  |  | RAPGEF1 |
|  |  |  |  |  |  |  |  | NTNG2 |
|  |  |  |  |  |  |  |  | BARHL1 |
|  |  |  |  |  |  |  |  | RP11-738I14.8 |
|  |  |  |  |  |  |  |  | GTF3C4 |
|  |  |  |  |  |  |  |  | TSC1 |
|  |  |  |  |  |  |  |  |  |
| **10p15.3** | 1365416 | 1636959 | 0.271543 |  |  |  |  | ADARB2 |
| **del** |  |  |  | (11/66) | 16.7 | (10/53) | 18.9 |  |
| **amp** |  |  |  | (7/66) | 10.6 | (7/53) | 13.2 |  |
|  |  |  |  |  |  |  |  |  |
| **10p14** | 10336388 | 10952891 | 0.616503 |  |  |  |  | CUGBP2 |
| **del** |  |  |  | (2/66) | 3.0 | (9/53) | 16.9 | C10orf31 |
| **amp** |  |  |  | (6/66) | 9.1 | (4/53) | 7.5 | UPF2 |
|  |  |  |  |  |  |  |  |  |
| **13q31.1** | 78952421 | 83938003 | 4.985582 |  |  |  |  | RBM26 |
| **del** |  |  |  | (12/66) | 18.2 | (19/53) | 35.8 | NDFIP2 |
| **amp** |  |  |  | (6/66) | 9.1 | (4/53) | 7.5 | SPRY2 |
|  |  |  |  |  |  |  |  | SLITRK1 |
|  |  |  |  |  |  |  |  |  |
| **13q31.2-31.3** | 86548316 | 89104192 | 2.555876 |  |  |  |  | SLITRK5 |
| **del** |  |  |  | (11/66) | 16.7 | (21/53) | 39.6 |  |
| **amp** |  |  |  | (5/66) | 7.6 | (5/53) | 9.5 |  |
|  |  |  |  |  |  |  |  |  |
| **13q13.3** | 90139305 | 90868110 | 0.728805 |  |  |  |  |  |
| **del** |  |  |  |  |  | (20/53) | 37.7 |  |
| **amp** |  |  |  |  |  |  |  |  |
|  |  |  |  |  |  |  |  |  |
| **14q12-q13.1** | 30042808 | 33265077 | 3.222269 |  |  |  |  | KIAA1333 |
| **del** |  |  |  | (5/66) | 7.6 | (10/53) | 28.9 | STRN3 |
| **amp** |  |  |  | (17/66) | 25.8 | (7/53) | 13.2 | NUBPL |
|  |  |  |  |  |  |  |  | AKAP6 |
|  |  |  |  |  |  |  |  | NPAS3 |
|  |  |  |  |  |  |  |  |  |
| **14q24.3-q31.1** | 78030767 | 79705600 | 1.674833 |  |  |  |  | NRXN3 |
| **del** |  |  |  | (8/66) | 12.1 | (12/53) | 22.6 |  |
| **amp** |  |  |  | (6/66) | 9.1 | (5/53) | 9.5 |  |
|  |  |  |  |  |  |  |  |  |
| **15q14-q15.1** | 37014833 | 37899611 | 0.884778 |  |  |  |  | C15orf54 |
| **del** |  |  |  | (14/66) | 21.2 | (15/53) | 28.3 | THBS1 |
| **amp** |  |  |  | (7/66) | 10.6 | (2/53) | 3.7 | THBS1 |
|  |  |  |  |  |  |  |  | GPR176 |
|  |  |  |  |  |  |  |  |  |
|  |  |  |  |  |  |  |  |  |
| **15q21.1** | 44175028 | 45398740 | 1.223712 |  |  |  |  | SQRDL |
| **del** |  |  |  | (14/66) | 21.2 | (17/53) | 32.1 |  |
| **amp** |  |  |  | (8/66) | 12.1 | (8/53) | 15.1 |  |
|  |  |  |  |  |  |  |  |  |
| **16p13.2** | 6859985 | 8241135 | 1.38115 |  |  |  |  | A2BP1 |
| **del** |  |  |  | (7/66) | 10.6 | (16/53) | 30.2 |  |
| **amp** |  |  |  | (9/66) | 13.6 | (3/53) | 5.6 |  |
|  |  |  |  |  |  |  |  |  |
| **16q12.2** | 51474818 | 53287244 | 1.812426 |  |  |  |  | CHD9 |
| **del** |  |  |  | (16/66) | 24.2 | (10/53) | 18.9 | RBL2 |
| **amp** |  |  |  | (11/66) | 16.7 | (11/53) | 20.7 | FTO |
|  |  |  |  |  |  |  |  | IRX3 |
|  |  |  |  |  |  |  |  |  |
| **17p13.2-p13.1** | 3899851 | 8524543 | 4.624692 |  |  |  |  | ZZEF1 |
| **del** |  |  |  | (16/66) | 24.2 | (17/53) | 32.1 | SPNS2 |
| **amp** |  |  |  | (4/66) | 6.1 | (3/53) | 5.6 | NLRP1 |
|  |  |  |  |  |  |  |  | AIPL1 |
|  |  |  |  |  |  |  |  | SLC13A5 |
|  |  |  |  |  |  |  |  | TXNDC17 |
|  |  |  |  |  |  |  |  | TP53 |
|  |  |  |  |  |  |  |  | NDEL1 |
|  |  |  |  |  |  |  |  |  |
| **18q21.33-q21.1** | 59063872 | 60196181 | 1.132309 |  |  |  |  | BCL2 |
| **del** |  |  |  | (5/66) | 7.6 | (14/53) | 26.4 | KDSR |
| **amp** |  |  |  | (12/66) | 18.2 | (2/53) | 3.7 | SERPINB3 |
|  |  |  |  |  |  |  |  | C18orf20 |
|  |  |  |  |  |  |  |  |  |
| **19q13.41-q13.43** | 58908108 | 62431059 | 3.522951 |  |  |  |  | CACNG6 |
| **del** |  |  |  | (12/66) | 18.2 | (7/53) | 13.2 | LILRB1 |
| **amp** |  |  |  | (8/66) | 12.1 | (2/53) | 3.7 | LOC284297 |
|  |  |  |  |  |  |  |  | NLRP11 |
|  |  |  |  |  |  |  |  | USP29 |
|  |  |  |  |  |  |  |  | AURKC |
|  |  |  |  |  |  |  |  |  |
| **21q21.1** | 19134684 | 21748820 | 2.614136 |  |  |  |  | PPIA |
| **del** |  |  |  | (6/66) | 9.1 | (17/53) | 32.1 | NCAM2 |
| **amp** |  |  |  | (7/66) | 10.6 | (1/53) | 1.8 |  |
|  |  |  |  |  |  |  |  |  |
| **21q21.3** | 27238687 | 27741249 | 0.502562 |  |  |  |  | ADAMTS5 |
| **del** |  |  |  | (7/66) | 10.6 | (15/53) | 28.3 |  |
| **amp** |  |  |  | (5/66) | 7.6 | (2/53) | 3.7 |  |

**Supplementary Table S3**

Clinicopathological information of non-small cell lung cancers for microRNA expression array (n=8)

| Sample# | Age | Race | Gender | Tumor size(cm) | Cell type | TNM stage | Smoking | Alcohol |
| --- | --- | --- | --- | --- | --- | --- | --- | --- |
| 847 | 72 | C | Male | 7.6 | SCC | IIB | Yes | Yes |
| 826 | 67 | C | Male | 4.0 | SCC | IIB | Yes | No |
| 829 | 80 | C | Male | 1.5 | ADC | IA | Yes | Yes |
| 740 | 65 | C | Male | 2.5 | ADC | IA | No | Yes |
| 775 | 58 | AA | Female | 3.5 | SCC | IB | Yes | Yes |
| 1216 | 50 | AA | Male | 4.0 | SCC | IIB | Yes | Yes |
| 1043 | 66 | C | Female | 2.8 | ADC | IIIA | No | No |
| 1135 | 61 | C | Male | 5.5 | SCC | IB | No | Yes |

AA: African-American, C: Caucasian, ADC: adenocarcinoma, SCC: squamous cell carcinoma

**Supplementary Table S4**

Technical validation of microRNA array data using Q-RT-PCR

|  | 829 | | 847 | | 1216 | | 1135 | |
| --- | --- | --- | --- | --- | --- | --- | --- | --- |
|  | ADC, Smoker | | SCC, Smoker | | SCC, Smoker | | SCC, Non-smoker | |
|  | Array | Q-RT-PCR | Array | Q-RT-PCR | Array | Q-RT-PCR | Array | Q-RT-PCR |
| *miR-205* |  | Up | Up | Up | Up | Up | Up | Up |
| *miR-296* |  | Up | Up | Up | Up | Up |  | Up |
| *miR-21* | Up | Up |  | Up |  | Up |  | Up |
| *miR-23b* |  | Up | Down | Down |  | Up | Down | Up |
| *miR-126* |  | Down | Down | Down | Down | Down | Down | Down |
| *miR-145* |  | Down | Down | Down | Down | Down | Down | Up |
| *miR-150* |  | Down |  | Down |  | Down |  | Up |
| *miR-31* |  | Up |  | Up |  | Up |  | Up |

Up: up-regulated in tumor, Down: down-regulated in tumor, Rosseta microRNA array results (array) in left column and Q-RT-PCR results (Q-RT-PCR) in right column

**Supplementary Table S5**

**Comparison between microRNA array results and Q-RT-PCR results in the technical validation set (n=8)**

|  | **Total**  Array results Q-RT-PCR  (Figure3a,n=8) (Fold change, n=8) | | **ADC**  Array results Q-RT-PCR  (Figure 3b,n=3) (Fold change, n=3) | | **SCC**  Array results Q-RT-PCR  (Figure 3c,n=5) (Fold change, n=5) | |
| --- | --- | --- | --- | --- | --- | --- |
| *miR-205* | Up | 6.89±1.32 | Up | 3.69±0.69 | Up | 9.28±1.22 |
| *miR-296* | Up | 2.40±0.81 | Up | 1.07±0.36 | - | 3.39±0.77 |
| *miR-21* | Up | 2.13±0.65 | Up | 2.06±1.35 | - | 2.20±0.51 |
| *miR-23b* | Down | 0.69±0.71 | - | -0.06±1.25 | - | 1.26±0.86 |
| *miR-126* | Down | -5.60±0.46 | Down | -4.57±0.45 | Down | -6.37±0.41 |
| *miR-145* | Down | -3.26±0.61 | Down | -3.51±0.49 | Down | -3.08±1.07 |
| *miR-150* | - | -0.58±0.69 | Up | 0.50±1.17 | - | -1.40±0.68 |
| *miR-31* | - | 4.54±0.80 | - | 2.47±0.76 | Up | 6.09±0.29 |

Up: up-regulated in tumor, Down: down-regulated in tumor, Fold change: Log_2_(Fold change value), Average ± Standard error

**Supplementary Table S6**

Comparison between microRNA array results and Q-RT-PCR results in the training set (n=18)

|  | Array results  in total  (Figure3a,n=8) | **ADC**  Array results Q-RT-PCR results (n=10)  (Figure 3b,n=3) Up Down | | | **SCC**  Array results Q-RT-PCR results (n=8)  (Figure 3c,n=5) Up Down | | |
| --- | --- | --- | --- | --- | --- | --- | --- |
| *miR-205* | Up | Up | ●●●○ | ○○○○○○ | Up | ●●●●●○○ | ○ |
| *miR-296* | Up | Up | ●●○○○ | ●○○○○ | - | ●●●●○○○ | ● |
| *miR-21* | Up | Up | ●●○○○○○○○ | ● | - | ●●●●○○○ | ● |
| *miR-23b* | Down | - | ●●○○○ | ●○○○○ | - | ●●●● | ●○○○ |
| *miR-126* | Down | Down |  | ●●●○○○○○○○ | Down |  | ●●●●●○○○ |
| *miR-145* | Down | Down |  | ●●●○○○○○○○ | Down | ● | ●●●●○○○ |
| *miR-150* | - | Up | ●○○○○○ | ●●○○ | - | ●○ | ●●●●○○ |
| *miR-31* | - | - | ●●●○○○○○○ | ○ | Up | ●●●●●○○○ |  |

Array results: From Figure 3a,b,c

Q-RT-PCR results: 3 ADCs and 5 SCCs (●), additional independent set of 7 ADCs and 3 SCCs (○)

**Supplementary Table S7**

Clinicopathological parameters of each cohort

| **factors** | **Technical validation set (n=8)** | **P value*** | **Training set (n=18)** | **P value**** | **Independent tumor cohort (n=114)** |
| --- | --- | --- | --- | --- | --- |
| Age | 64.9 | N.S. | 66.2 | N.S. | 65.7 |
| Race (AA/C/Other) | 2/6/0 | N.S. | 3/15/0 | N.S. | 22/88/4 |
| Gender (Female/Male) | 2/6 | N.S. | 4/14 | N.S. | 62/52 |
| Histology (ADC/SCC) | 3/5 | N.S. | 10/8 | N.S. | 69/45 |
| Differentiation(Well/Mod/Poor) | 2/4/2 | N.S. | 2/8/8 | N.S. | 22/62/30 |
| TNM stage (I/II/III/IV) | 3/2/3/0 | N.S. | 4/9/3/2 | N.S. | 63/27/19/5 |
| TNM stage (I/II-IV) | 3/5 | N.S. | 4/14 | **0.011** | 63/51 |
| Smoking history (Yes/No) | 5/3 | N.S. | 10/8 | N.S. | 67/47 |
| Alcohol history (Yes/No) | 6/2 | N.S. | 14/4 | N.S. | 73/41 |

AA: African-American, C: Caucasian, ADC: adenocarcinoma, SCC: squamous cell carcinoma, N.S.: not significant

*Technical validation set (n=8) versus Training set (n=18) (Fisher’s exact test or Student’s t-test, two sided)

**Trainig set (n=18) versus independent tumor cohort (n=114)(Fisher’s exact test or Student’s t-test, two sided)

If significant displayed bold.

**Supplementary Table S8**

Correlation of miR expression with clinicopathological parameters in validation cohort (n=114)

| microRNA | *miR-21* | *miR-23b* | *miR-31* | *miR-126* | *miR-145* | *miR-150* | *miR-205* | *miR-296* |
| --- | --- | --- | --- | --- | --- | --- | --- | --- |
| Age (>60 vs ≤60) | N.S. | N.S. | N.S. | N.S. | N.S. | N.S. | N.S. | N.S. |
| Race (AA vs non-AA) | N.S. | N.S. | N.S. | N.S. | N.S. | N.S. | N.S. | N.S. |
| Gender (Female vs Male) | N.S. | N.S. | N.S. | N.S. | N.S. | N.S. | N.S. | N.S. |
| Smoking history (Yes vs No) | N.S. | N.S. | N.S. | **0.030** | N.S. | N.S. | N.S. | N.S. |
| Alcohol history (Yes vs No) | N.S. | N.S. | N.S. | N.S. | N.S. | N.S. | N.S. | N.S. |
| Histology (ADC vs SCC) | N.S. | N.S. | N.S. | N.S. | N.S. | N.S. | **<0.001** | N.S. |
| Differentiation (Well/Mod vs Poor) | N.S. | N.S. | N.S. | N.S. | N.S. | N.S. | N.S. | N.S. |
| T stage (T1 vs T2/T3/T4) | N.S. | N.S. | N.S. | N.S. | N.S. | N.S. | N.S. | N.S. |
| N stage (N0 vs N1/N2) | N.S. | **0.047** | N.S. | N.S. | N.S. | N.S. | N.S. | N.S. |
| TNM stage (I vs II/III/IV) | N.S. | N.S. | N.S. | N.S. | N.S. | N.S. | **0.034** | N.S. |

AA: African-American, ADC: adenocarcinoma, SCC: squamous cell carcinoma, N.S.: not significant, P value: Student’s t-test, If significant displayed bold.

**Supplementary Table S9**

The results of copy number variation analysis for *miR-21* locus (17q23.2)

**Amplification (>2.5) Deletion (<1.7)**

| Marker | **Chromosome** | **Position** | **Normal** | **Tumor** | **P value** | **Normal** | **Tumor** | **P value** |
| --- | --- | --- | --- | --- | --- | --- | --- | --- |
| **SNP_A-1515788** | **17** | 59136652 | **2** | **9** | **0.059** | **23** | **15** | **0.215** |
| **SNP_A-1507433** | **17** | 60710260 | **3** | **12** | **0.030** | **23** | **15** | **0.215** |

Position: UCSC Genome Bioinformatics Feb.2009 (GRCh37/hg19) Normal: n=119, Tumor: n=119, P value: Fisher’s exact test, two-sided N.S.: not significant, The nearest markers of *miR-21* were indicated by bold characters.

**Supplementary Table S10**

The results of copy number variation analysis for *miR-23b* locus (9q22.32)

**Amplification (>2.5) Deletion (<1.7)**

| Marker | **Chromosome** | **Position** | **Normal** | **Tumor** | **P value** | **Normal** | **Tumor** | **P value** |
| --- | --- | --- | --- | --- | --- | --- | --- | --- |
| SNP_A-1516041 | 9 | 96820184 | 5 | 12 | N.S. | 32 | 23 | N.S. |
| SNP_A-1516182 | 9 | 97560204 | 10 | 11 | N.S. | 31 | 21 | N.S. |
| **SNP_A-1507358** | **9** | **97764492** | **8** | **18** | **0.060** | **31** | **13** | **0.004** |
| **SNP_A-1518385** | **9** | **97936435** | **7** | **19** | **0.021** | **30** | **12** | **0.004** |
| SNP_A-1508003 | **9** | 98112299 | **5** | 28 | <0.001 | 29 | 14 | 0.018 |
| SNP_A-1510002 | 9 | 99113188 | 7 | 28 | <0.001 | 33 | 16 | 0.010 |

Position: UCSC Genome Bioinformatics Feb.2009 (GRCh37/hg19) Normal: n=119, Tumor: n=119, P value: Fisher’s exact test, two-sided N.S.: not significant, The nearest markers of *miR-23b* were indicated by bold characters.
